# Early hominin arrival in Southeast Asia triggered the evolution of major human malaria vectors

**DOI:** 10.1038/s41598-026-35456-y

**Published:** 2026-02-26

**Authors:** Upasana Shyamsunder Singh, Ralph E. Harbach, Jeffery Hii, Moh Seng Chang, Pradya Somboon, Anil Prakash, Devojit Sarma, Ben S. Broomfield, Katy Morgan, Sandra Albert, Aparup Das, Yvonne-Marie Linton, Jane M. Carlton, Catherine Walton

**Affiliations:** 1 Department of Earth and Environmental Sciences, School of Natural Sciences, University of Manchester, Manchester, UK; 2 Present Address: Department of Biological Sciences, Vanderbilt University, Nashville, USA; 3 Department of Science, Natural History Museum, Cromwell Road, London, UK; 4 College of Public Health, Medical and Veterinary Sciences, James Cook University, North Queensland, Australia; 5 Department of Community Medicine & Public Health, University Malaysia Sarawak, Sarawak, Malaysia; 6 Center of Insect Vector Study, Department of Parasitology, Faculty of Medicine, Chiang Mai University, Chiang Mai, Thailand; 7 ICMR-Regional Medical Research Centre, Dibrugarh, Assam India; 8 ICMR-National Institute for Research in Environmental Health, Bhopal, India; 9 Indian Institute of Public Health Shillong, Shillong, Meghalaya India; 10 ICMR-National Institute of Research in Tribal Health, Jabalpur, Madhya Pradesh India; 11 Walter Reed Biosystematics Unit, Smithsonian Museum Support Center, Suitland, MD USA; 12 Department of Entomology, Smithsonian Institution – National Museum of Natural History, Washington, DC USA; 13 One Health Branch, Walter Reed Army Institute of Research, Silver Spring, MD USA; 14 Johns Hopkins Malaria Research Institute, Bloomberg School of Public Health, Baltimore, USA

**Keywords:** Ecology, Ecology, Evolution, Genetics, Zoology

## Abstract

Some species of the Leucosphyrus Group of *Anopheles* mosquitoes in Southeast Asia are highly anthropophilic and efficient vectors of human malaria parasites, while others primarily feed on non-human primates (NHP) and transmit NHP malaria parasites. The evolutionary history of this group, particularly the origin of anthropophily, was studied using phylogenomic analysis of 2,657 high-confidence nuclear single-copy orthologous genes and 13 mitochondrial protein coding genes from 40 individuals of 11 species. Molecular dating and ancestral state reconstruction revealed that monkey-feeding is ancestral with speciation of monkey-feeding species dating to the Pliocene within Sundaland (Malay peninsula, Borneo, Sumatra and Java) which was covered in tropical rain forests during this period. Although less parsimonious alternatives cannot be excluded, molecular dating, ancestral state reconstruction and reticulation analysis indicated that anthropophily most likely evolved once, involving adaptive introgression, in the early Pleistocene in Sundaland, giving rise to multiple descendent anthropophilic species. Such early origination of anthropophily must necessarily have been in response to the arrival of early hominins (*Homo erectus*) rather than anatomically modern humans, likely associated with loss and fragmentation of rainforests during the early Pleistocene. The early origination of anthropophily also provides independent non-archaeological evidence supporting the limited fossil record of early hominin colonization in Southeast Asia around 1.8 Mya.

**Supplementary Information:**

The online version contains supplementary material available at 10.1038/s41598-026-35456-y.

## Introduction

Mosquito-borne diseases present a significant burden on human health, with malaria alone causing an estimated 249 million cases and 608,000 deaths worldwide in 2022^1^. The propensity of mosquitoes of a particular species to feed on humans (anthropophily) is the primary factor influencing their potential to spread pathogens that cause disease^2–7^. Although mosquitoes can be opportunistic in their host selection (e.g^8,9^.,, many species display varying degrees of host specificity^10,11^. Understanding the evolutionary origins of anthropophily and the circumstances that triggered its development can provide critical insights into mitigating the impacts of novel diseases due to mosquito-borne pathogens.

**Fig. 1:**
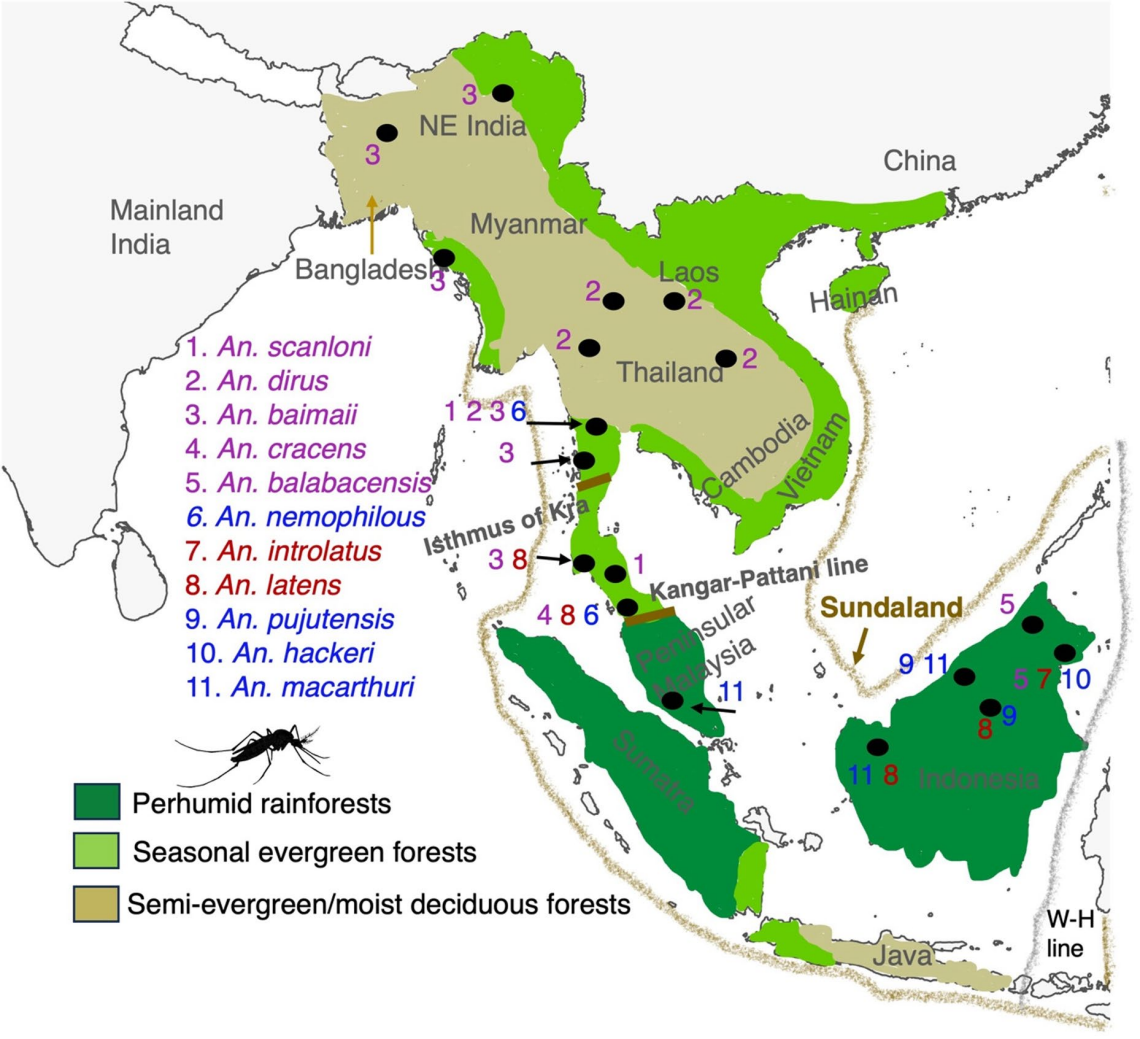
Map representing the distributions of specimens collected in Southeast Asia. Shading indicates the present-day distributions of forest types in mainland and insular Southeast Asia, adapted from Morley^55^. Black dots on the map represent collection sites. The number adjacent to the dots represents individual species collected from that site according to the species list on the left. The color of the numbers and species names indicate distinct blood-feeding behaviors; blue–NHP feeding, red–mixed-feeding, purple–human feeding, derived from published literature (listed in Supplementary Table S1). The brown outer line represents the outline of the exposed Sunda Shelf at the Last Glacial Maxima (currently 120 m below sea level)^57^. The two short brown lines represent biogeographic barriers: the Kangar-Pattani line in the south and the Isthmus of Kra in the north. The Isthmus of Kra and the W-H (Wallace-Huxley) line (grey line) in the east mark the boundaries of Sundaland. The map was created using QGIS v.3.28 (http://www.qgis.org).

The *Anopheles leucosphyrus* group (hereafter, Leucosphyrus Group) comprises 20 recognized mosquito species in Southeast Asia (SE Asia)^12–15^. These species exhibit intrinsic differences in host preference, as demonstrated by host attraction experiments, blood-meal analysis, and variation in transmission of human and non-human primate (NHP) malarias (Supplementary Table S1)^13,16–19^. Notably, several species are highly anthropophilic and extremely efficient vectors of human malaria parasites. These include *An. dirus*,* An. baimaii*, and *An. scanloni* of the Dirus Complex found in mainland SE Asia, and *An. balabacensis* of the Leucosphyrus Complex from Borneo (Sabah and Kalimantan)^17,20–23^ (Supplementary Table S1). Conversely, species such as *An. macarthuri*, *An. pujutensis*, and *An. hackeri* blood-feed only in the forest canopy on NHP, including monkeys, gibbons, and orangutans, transmitting NHP malaria parasites (Fig. 1, Supplementary Table S1)^24–27^. *Anopheles nemophilous* (of the Dirus complex), *An. latens*, and *An. introlatus* (of the Leucosphyrus Complex) are less host-specific, feeding on both NHPs in the canopy and humans on the ground, apparently driven by host availability (Supplementary Table S1). As host choice experiments most often compared humans on the ground, to monkeys in the canopy, it is not possible to separate a tendency to seek hosts on the ground rather than in the canopy as a distinct trait^23,28,29^ (Supplementary Table SI).

The establishment of anthropophily in multiple species of the Leucosphyrus Group could be attributed to the trait evolving independently multiple times following the arrival of anatomically modern humans in SE Asia 76,000–63,000 years ago^30,31^. Alternatively, anthropophily may have evolved once in an ancestral species, possibly in response to the colonization of SE Asia by early hominins. Conservative estimates place *Homo erectus* in China at least 1.6–1.7 million years ago (Mya), and possibly as long ago as 2.4 Mya^32^. However, the timeline of hominin colonization southwards into SE Asia remains contentious. Recent reports suggest that hominins may have arrived in Java between 1.3^33^ and 1.8 Mya^34^. Increased aridity during the Late Pliocene and Early Pleistocene, particularly during periodic glacial periods, is considered to have resulted in the formation of a north-to-south corridor of seasonal forests and grasslands^35^, that facilitated early hominin migration through SE Asia into Java^36^. We used phylogenomics and analyses of trait evolution in the Leucosphyrus Group to characterize the evolutionary history of these mosquitoes in relation to historical environmental changes and host preference. Our findings offer independent, non-archaeological evidence for the timing and location of the early hominin colonization of SE Asia, providing new perspectives on the co-evolution of mosquitoes and their hosts.

## Results and discussion

### Genome-scale phylogenies reveal reticulate evolution in the Leucosphyrus Group

**Fig. 2:**
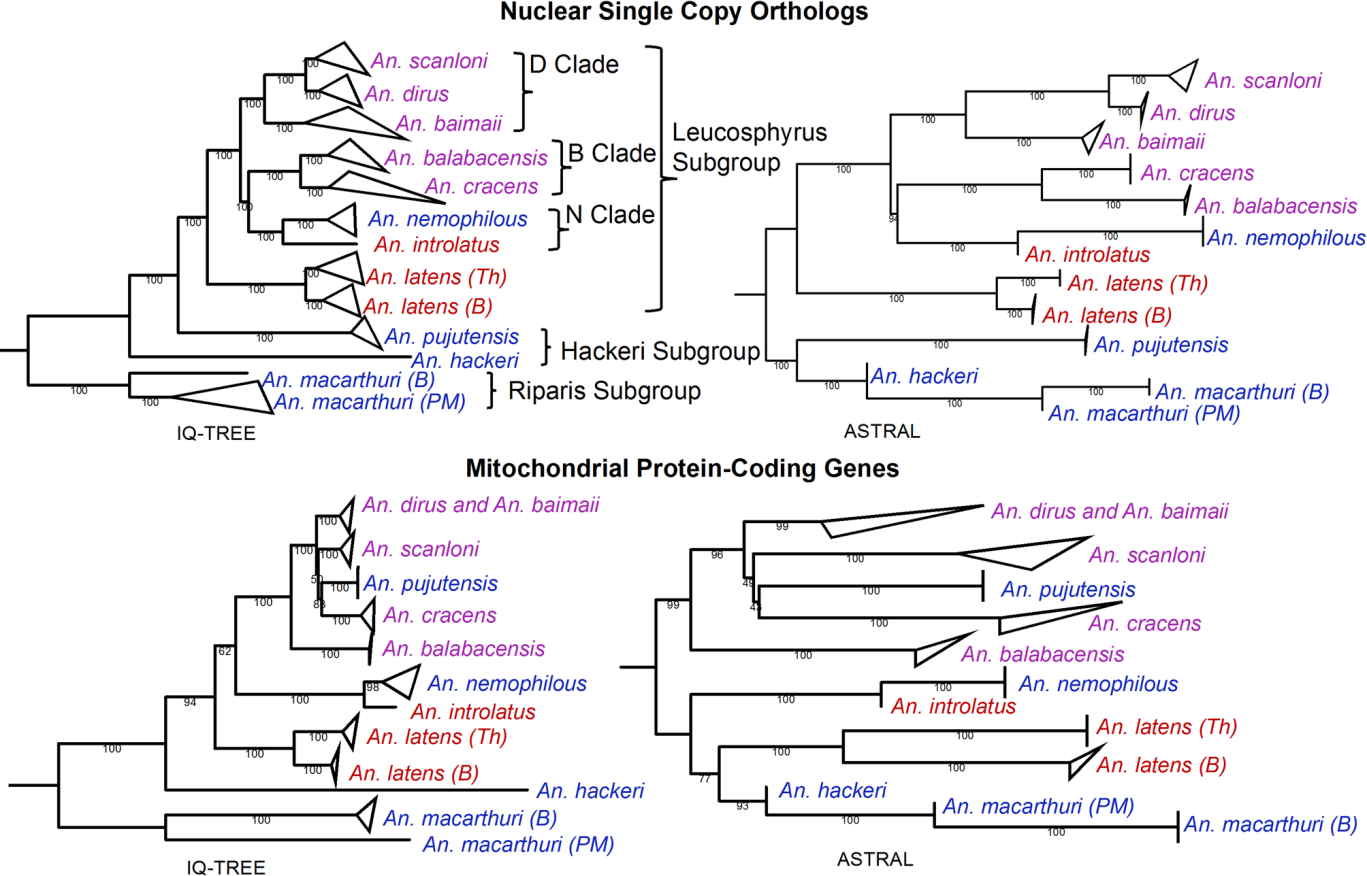
**Genome-scale phylogenies of the Leucosphyrus Group.** Trees were constructed using concatenation-based (IQ-TREE) (right) and ASTRAL (left) approach. The nuclear trees (top) were constructed using 2,657 single copy orthologs. The mitochondrial trees (bottom) were constructed using 13 protein-coding genes. Numbers on the branches denote bootstrap values. Species names are color-coded according to feeding preferences as in Fig. 1 and table S1. Based on the morphological classification by Sallum et al.^17^, the Leucosphyrus Group comprises the Riparis, Hackeri and Leucosphyrus Subgroups. The Leucosphyrus Subgroup is further divided into the Dirus Complex (*An. baimaii*, *An. dirus*, *An. scanloni*, *An. cracens* and *An. nemophilous*) and the Leucosphyrus Complex (*An. balabacensis*,* An. latens* and *An. introlatus*)^17^. The morphological classification and phylogenetic classification do not agree therefore, based on the nuclear phylogeny, we classify *An. dirus*, *An. baimaii* and *An. scanloni* as the Dirus Clade (D Clade), *An. balabacensis* and *An. cracens* as the *Balabacensis* Clade (B Clade), and *An. introlatus* and *An. nemophilous* as the Nemophilous Clade (N Clade). Different population of *An. latens* and *An. macarthuri* are denoted by: Th–Thailand, B–Borneo and PM–Peninsular Malaysia. The color of the species names indicates distinct blood-feeding behaviors; blue–NHP feeding, red–mixed-feeding, purple–human feeding.

To elucidate the evolutionary history of the Leucosphyrus Group, we sequenced 38 individual mosquitoes of 11 species: *An. dirus* (*n* = 5), *An. baimaii* (*n* = 6), *An. scanloni* (*n* = 6), *An. cracens* (*n* = 1), *An. nemophilous* (*n* = 2), *An. introlatus* (*n* = 1), *An. balabacensis* (*n* = 5), *An. hackeri* (*n* = 1), *An. latens* (*n* = 5), *An. macarthuri* (*n* = 3), and *An. pujutensis* (*n* = 3). These data were supplemented with the publicly available genomes of *An. dirus*^37^ and *An. cracens*^38^. Many of these species are particularly challenging to collect, for example, involving sampling larvae from animal wallows deep in the forest and from remote locations. Specimens of the 11 species studied here were accumulated over several years (from 1992 to 2020) and include all species of the Leucosphyrus Group from Sundaland and Indochina except, for logistical reasons, those restricted to Sumatra and the Philippines. The 11 species studied here include members of all three Subgroups (Leucosphyrus, Riparis and Hackeri) as originally proposed by Peyton and later adopted by Sallum et al.^17^. They also represent all three blood-feeding behaviors (human, NHP and mixed human-NHP) (Fig. 1, Supplementary Table S2). Orthology inference using *An. dirus* and an outgroup species, *An. farauti*, identified 2,657 high-confidence nuclear single-copy orthologous genes (nSCOs) across 40 genomes. Phylogenetic reconstructions were performed using coalescent-based summary analyses with ASTRAL^39^ and maximum-likelihood (IQ-TREE)^40^ analyses on 2,657 nuclear and 13 mitochondrial protein-coding DNA sequences (Fig. 2).

There were some inconsistencies between the resultant nuclear and mitochondrial phylogenies (Fig. 2). *Anopheles dirus* and *An. baimaii*, though distinct from each other in the nuclear phylogenies, are indistinguishable in mitochondrial phylogenies, consistent with mitochondrial introgression from *An. baimaii* to *An. dirus* as previously reported^41^. *Anopheles pujutensis* (Hackeri Subgroup) is placed within the Dirus Complex in the mitochondrial phylogenies which is very different from its placement with other NHP-feeding species in the nuclear phylogenies. We infer this to be the result of older mitochondrial introgression from a member of the Dirus Complex into *An. pujutensis* (Fig. 2). Incomplete lineage sorting cannot be an explanation in either of these two cases since there is no evidence of incomplete lineage sorting in the nuclear phylogeny with all individuals within a species forming distinct clades and given that lineage sorting would be even faster for mtDNA with its lower effective population size. *Anopheles cracens* is also differently placed between the mitochondrial and nuclear trees (Fig. 2). Due to this mitochondrial introgression, we focused subsequent analyses on the nuclear data.

The tree topologies for each genomic region (nuclear or mitochondrial) were consistent across phylogenetic methods, except for the placement of a midpoint root among *An. macarthuri*, *An. hackeri*, and *An. pujutensis*, indicating variation in branch length estimation between ASTRAL and IQ-TREE methods. Discordance between coalescent-based (ASTRAL) and concatenation-based (IQ-TREE) methods is commonly observed and can be attributed to multiple causes, including differing evolutionary histories among loci, rate variation among lineages, or model misspecification^42,43^. To investigate these discrepancies, we used PhyloNet^44^ to construct phylonetworks. As the number of reticulations increased from one to three, the likelihood of the phylonetworks increased (Supplementary Fig. S1). Although more than three reticulations might be likely, further exploration was limited by computational power. All networks with 1–3 reticulations indicated either substantial introgression and/or incomplete lineage sorting of nuclear loci between lineages leading to *An. pujutensis*,* An. hackeri*, and *An. macarthuri* and some introgression between these lineages and more recently derived species (Supplementary Fig. S1). The introgression or incomplete lineage sorting may significantly contribute to discrepancies in the placement of the midpoint root (Fig. 2).

Given the large number of nuclear loci used, general congruence of topology between different methods of phylogenetic reconstruction, and high levels of bootstrap support for most branches, the interpretations of evolutionary history below utilise the nuclear phylogenies that we consider to best represent the species history. In making these interpretations we consider how introgression and/or incomplete lineage sorting could contribute to uncertainty.

### Evolutionary timeline and geographical origin of mosquito feeding preference

Overall, the nSCO phylogenies challenge the current morphology-based taxonomic classification^17^. While there is support for the monophyly of the Leucosphyrus Subgroup, the phylogenies do not support the monophyly of the Dirus and Leucosphyrus Complexes within this Subgroup (Fig. 2). Despite uncertainty in the order of basal branching due to introgression, the high topological concordance of the nuclear phylogenies provides a robust phylogenetic framework to study the evolution of host preference for species of the Leucosphyrus Group.

**Fig. 3:**
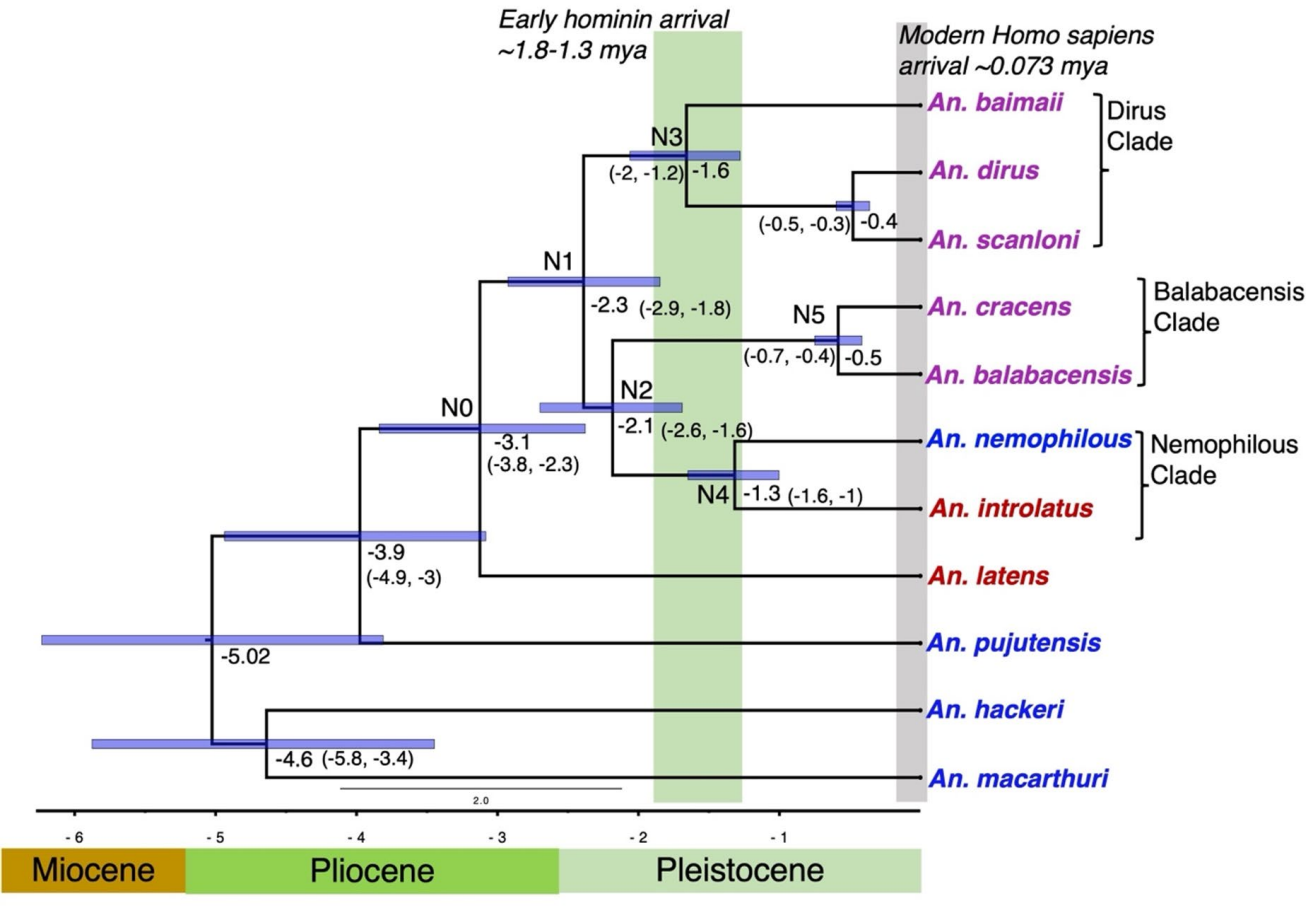
Chronogram of the Leucosphyrus Group using a Bayesian approach. The divergence dating analysis was carried out using 25 clock-like genes and the coalescent species tree estimation method. The estimated divergence times are indicated by the numbers at the nodes; the blue error bars denote 95% highest posterior densities, and the numbers in parentheses are confidence intervals. The green vertical shading represents the time interval proposed for the arrival of early hominins in Southeast Asia based on fossil data between 1.3^33^ and 1.8 million years ago^34^. The vertical grey line represents the time of arrival of anatomically modern humans^30^. The color of the species names indicates distinct blood-feeding behaviors; blue–NHP feeding, red–mixed-feeding, purple–human feeding. (see Fig. 1 and Supplementary Table S1).

The interpretations below depend on the estimates of divergence times, so we have taken several steps to make the dating as reliable as possible. Incomplete linage sorting in rapidly diverging species and the large effective population sizes expected of mosquitoes, could lead to over estimation of divergence time due to coalescence times being much older than species divergence times^45^. Further, introgression as inferred here could also introduce error into dating. To address these issues, divergence times were estimated under the multi-species coalescent^46^. To minimize the issues of rate heterogeneity among genes and topological conflict of gene trees with the species tree, divergence times were estimated using a subset of 25 genes that exhibited the most clock-like behaviour (had the lowest root-to-tip variance) and that had a high degree of conformity to the species tree, evaluated using SortaDate^47^. Use of this reduced number also served to make the analysis computationally tractable. This approach of using reduced, clock-like datasets has been widely applied in molecular dating studies (e.g^48–50^.,. An advantage of this approach is that it enables a strict molecular clock (Fig. 3) to be used for dating which gives more precise estimates than a relaxed clock (Fig. S3). For this reason, these results are presented below, but divergence dating using a relaxed-clock model with the same 25 nuclear genes generated very similar divergence estimates (Fig. S2).

Due to lack of fossil or geological calibration points, divergence dating here relies on the use of a molecular clock. As no estimates of mutation rate are available for *Anopheles*, we applied the *Drosophila melanogaster* mutation rate of 2.8 × 10⁻⁹ mutations per site per generation^51^. While mutation rates differ between species, they are not expected to vary substantially between *Drosophila* and *Anopheles* as these reasonably closely related Dipteran taxa share many similar life history traits (including generation times and population sizes), metabolic rates, recombination rates and genomic architecture. Potential error associated with the application of the *Drosophila* mutation rate to *Anopheles* should not be forgotten in the discussion below, but it is expected to be minimal, as reflected by the widespread use of the *Drosophila* mutation rate for *Anopheles* species^52–54^. The mutation rate is expected to be very similar to the substitution rate (the rate at which new mutations are passed on to future generations) for non-coding regions where new mutations are largely expected to be neutral. By contrast, the substitution rate in coding data is expected to be significantly lower than the mutation rate and not accounting for this would lead to underestimation of divergence times^55^. Thawornwattana et al.^52^ have demonstrated in *Anopheles gambiae* that divergence rate in the coding regions is 0.524X lower than in non-coding regions, so a scaling factor of 0.524 has been applied here to the mutation rate to account for the use of only coding data (Fig. 3).

**Fig. 4:**
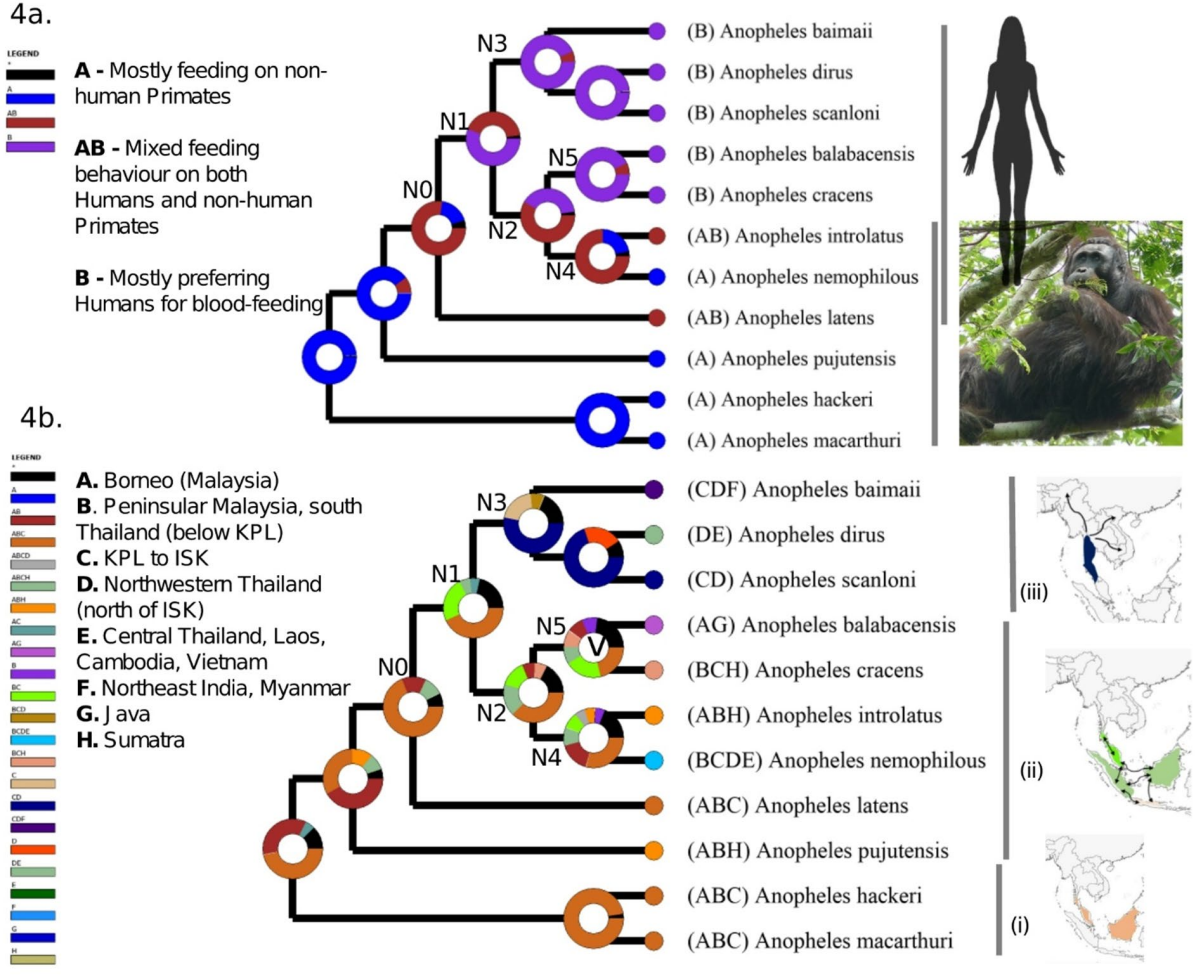
Reconstruction of ancestral states for blood-feeding behavior and biogeography. **(4a)** Trait evolution analysis of host preference for blood-feeding. The node N0 represents a switch from feeding only on NHPs in the canopy to also feeding on humans on the ground. Node N1/N2 represents the earliest inferred switch to a preference for feeding on humans. Node N3 represents the ancestor of the Dirus Clade; N4–ancestor of the Nemophilous Clade, and N5–ancestor of the Balabacensis Clade. **(4b)** Reconstruction of the ancestral states for biogeography. This analysis shows dispersal at every node except for node N5 where “V” inside the pie denotes a vicariance event. The schematic maps on the right (**i–iii**) show dispersal within the Leucosphyrus Group for the corresponding part of the phylogeny, where (**i**) the orange color shows that peninsular Malaysia and Borneo is the ancestral range of monkey-feeding groups, (**ii**) green shows subsequent dispersal between Peninsula Malaysia, Sumatra, Borneo, and Java followed by (**iii**) shown in blue, dispersal of the Dirus Clade to and across the Indochinese region. Black arrows indicate direction of dispersal. The map was created using QGIS v.3.28 (http://www.qgis.org).

To test the reliability of the nuclear molecular clock used above we also conducted divergence dating using the mitochondrial *COI* gene as the molecular clock rate of 2.3% divergence per million years has been well established for this gene by Brower^56^. Although direct comparisons are difficult due to the issue of mitochondrial introgression, the mitochondrial chronogram (Fig. S3 ) estimates divergence dates that are highly consistent with the nuclear chronogram. Notably, a key divergence date at node N0, which is associated with a transition from the ancestral state, has highly consistent dates and confidence intervals in the mitochondrial (2.98 Mya (95% CI: 3.4–2.5 Mya) (Fig. S3) and nuclear 3.1Mya (95% CI: 3.8–2.3 Mya) (Fig. 3) chronograms. The use of a carefully curated set of genes and the congruency between the mitochondrial and nuclear clocks give considerable confidence in the resulting chronogram, which was used to estimate divergence times (Fig. 3), and to reconstruct the ancestral states for host preference (Fig. 4a) and biogeographical distribution (Fig. 4b) using Reconstruction of Ancestral States (RASP) v4^57^. Below, we integrate these lines of evidence, together with information on historical environmental change, to infer the evolutionary history of the group. The use of molecular clocks, rather than a fossil or geological calibration point, necessarily introduces some uncertainty into dating so interpretations of lineage-specific traits below are made with this caveat in mind.

### Ancestors of the leucosphyrus group were feeding on non-human primates in Sundaland

Extensive introgression and/or lineage sorting among *An. macarthuri*, *An. hackeri*, and *An. pujutensis* complicates the precise estimation of divergence times for these species. Despite uncertainty in the relationships between these three species we can infer with confidence that these NHP-feeding species are basal and diverged during the early Pliocene (5.3–3.6 Mya) (Fig. 3). Ancestral trait reconstruction for host preferences (Fig. 4a) corroborates the expectation that NHP-feeding is the ancestral state. Additionally, biogeographical reconstruction (Fig. 4b) indicates these three species originated in Sundaland (Fig. 1), encompassing present-day Borneo, peninsular Malaysia, peninsular Thailand below the Isthmus of Kra, Sumatra, and the currently submerged Sunda Shelf. During this period, the region was covered by extensive permanently humid (perhumid) rainforests^58,59^, providing ample opportunity for specialization in feeding on NHPs in the forest canopy.

### Speciation associated with Plio-Pleistocene environmental change

The Pliocene and early Pleistocene were characterized by increasingly cooler and drier global climates^60,61^. It is during this period, characterized by extensive environmental change, that the Leucosphyrus Subgroup emerged, with the ancestral species *An. latens* diverging at node N0, around 3.1 Mya [95% CI: 3.8–2.3 Mya] (Fig. 3). Subsequently, four divergence events (nodes N1–N4, Fig. 3) occurred in the early to mid-Pleistocene (2.3–1.3 Mya) within Sundaland (Fig. 4b), with divergence at N1 giving rise to the Dirus Clade (comprising *An. dirus*,* An. baimaii and An. scanloni*) and divergence at N2 giving rise to the Nemophilous (*An. nemophilous* and *An. introlatus*) and Balabacensis (*An. balabacensis* and *An. cracens*) Clades (Figs. 2 and 3). It has long been viewed that eustatic sea-level changes were the major driver of diversification during the Pleistocene, with repeated splitting and reformation of the Sundaland landmass^58,59,62^. This view has changed with the recognition of subsidence of the Sunda Shelf during the Pleistocene, which means that it must have been exposed as a single landmass continuously until 400,000 years ago^63^. It is only after 400,000 years that Java, Sumatra, Borneo, and Indochina–Peninsular Malaysia were separated by elevated sea levels during interglacial periods (Fig. 1). Consequently, throughout the late Pliocene and most of the Pleistocene the balance of evidence favors a seasonal corridor that extended from Indochina southwards through central Sundaland, featuring more open and seasonal forests and potentially including grasslands particularly during drier glacial periods^35,64–70^. Therefore, we propose that the apparent burst of speciation occurring at nodes N0, N1–N4 within Sundaland at this time involved adaptation to novel forest types, hosts associated with these new habitats, or vicariance involving repeated fragmentation of perhumid rainforests. Since bi-directional dispersal was detected (Fig. 4b) between present-day peninsular Malaysia, Sumatra, Borneo, and Java, it indicates that there may have been limited periods of forest connectivity across Sundaland^59^. The recent divergence of *An. cracens* and *An. balabacensis*, which are restricted to peninsular Malaysia and Borneo, respectively, at node N5 around 0.5 Mya [95% CI: 0.7–0.4 Mya] (Figs. 3 and 4b), is attributed to vicariance by the RASP v4 analysis, likely due to the formation of separate landmasses during interglacial periods.

### Crossing the barrier of the isthmus of Kra and origin of the Dirus clade

The Isthmus of Kra (ISK) marks a significant biogeographical boundary for many forest species, including birds^67^, due to the seasonally dry climates in lowlands further north of ISK, in Thailand^59^. While *An. nemophilous* is found both north and south of the ISK barrier, its distribution is confined to seasonal evergreen forests south of the ISK, and above the Kangar-Pattani line (K-PL) and patches found on the Thai-Cambodia border (Fig. 1)^20^. Speciation of the ancestor of the Dirus Clade likely involved adaptation to the seasonal forests north of ISK that enabled it to cross this biogeographical boundary. This ancestral species migrated northward, where it subsequently gave rise to at least three species (*An. dirus*, *An. baimaii*, and *An. scanloni*), with *An. dirus* and *An. baimaii* dispersing extensively eastwards and westwards, respectively (Fig. 4b and c(iii)), becoming major vectors of human malaria parasites across much of Indochina^68^.

### Two-stage transition to feeding on humans in Sundaland

The shift away from strict canopy feeding on NHPs is inferred to have begun in the Late Pliocene (Fig. 3), with the emergence of the Leucosphyrus Subgroup (node N0, Figs. 3 and 4a). The late Pliocene is characterized by the transition from perhumid to seasonal and open forest types and increased savannah^59,65,71^. During this period a diverse assemblage of terrestrial mammals adapted to these novel habitats is likely to have inhabited forest-savannah mosaics in Sundaland based on fossil evidence from Early Pleistocene sites^66^. Unlike its canopy-feeding ancestors, the basal species of this Subgroup, *An. latens*, readily feeds on humans and other mammals on the ground as well as NHPs in the canopy^24,26,65–72^ (Supplementary Table S1). The increased abundance of ground dwelling host species during the late Pliocene could therefore have trigged an adaptive evolutionary innovation in host-seeking behavior of *An. latens* involving a willingness to seek hosts on the ground. This evolutionary transition could have acted as the bridge to human-feeding behaviour.

### Evolution of vectors of malaria parasites in response to early hominin colonization of Southeast Asia

Mosquitoes employ multiple senses to track their hosts, but evolutionary changes in olfactory genes, particularly those involving the fine tuning of olfactory receptors through modification of their expression and specificity, are crucial for developing a preference for human body odor^73^. Large numbers of odorants and olfactory genes are involved in host specificity and genomic studies in *Ae. aegypti*^74^, *An. farauti*^75^, *Culex pipiens*^76^ and *An. gambiae*^77^ reveal that multiple genetic changes at these and other genes are required for the evolution of anthropophily, i.e. a strong, evolved preference for human blood. It is not surprising therefore that anthropophily is uncommon amongst the ~ 3500 known mosquito species^78^. It is therefore more parsimonious to consider that anthropophily within a taxon has a single origin. Accordingly, it is improbable that there were multiple independent switches to anthropophily in the human-preferring species of the Dirus and Balabacensis Clades, which diverged around 1.3–0.5 Mya (Fig. 3). Even taking into account some uncertainty in the molecular clock used for these divergence estimates these clades far predate the arrival of anatomically modern humans in SE Asia 76,000–63,000 years ago^30,31^. We therefore reject with confidence the hypothesis that anthropophily in the Leucosphyrus Subgroup evolved in response to the arrival of modern humans in SE Asia.

Using the same molecular clock rate as applied in this study and a species tree, anthropophily would be inferred to have evolved ~ 509,000–61,000 years ago in the lineage leading to the major African malaria vectors, *An. gambiae* and *An. coluzzii*^52^. This would date it well before the development of agriculture several thousand years ago as has been previously suggested^79^. Since *An. gambiae* originates in West African forests^80^ the switch to anthropophilly may instead have occurred in response to modern humans entering this forested region ~ 150,000 years ago^81^. The emergence of anthropophily in the domestic form of *Aedes aegypti*^74^ and the molestus ecotype of *Culex pipiens*^7^, both date to within the last 10,000 years, apparently in response to growing human populations and environmental change. Together these studies indicate that the abundance of a novel host source is a key requisite for triggering the evolution of anthropophily. The Fig. 3 indicates that anthropophily in the Leucosphyrus Subgroup emerged much earlier than in other anthropophilic mosquito species. If a strictly bifurcating tree were assumed, anthropophily could have evolved either: by the time of node N1 [95% CI: 2.9–1.8 Mya] and subsequently been lost in the lineage leading to *An. nemophilous* and *An. introlatus*; or evolved twice along the lineages leading to the Dirus Clade (N3, 1.6 Mya [95% CI: 2.0–1.2 Mya]) and the Balabacensis Clade (N5, 0.5 Mya [95% CI: 0.7–0.4 Mya]). However, the short internode distance between N1 and N2, their highly overlapping confidence intervals for divergence times, and the low level of bootstrap support for N2 in the ASTRAL phylogenetic tree (Fig. 2) indicate that this period of speciation history more likely corresponds to a phylogenetic network rather than a bifurcating tree. A network would result from introgression among incipient species and/or incomplete lineage sorting between N1 and N2. This is further supported by both the detection of reticulation from our reticulation analysis (Fig. S1) and mitochondrial introgression (Fig. 2).

Numerous genomic studies have shown the importance of introgression in promoting species adaptation to novel environments i.e. adaptive introgression^82^, including during speciation within the *An. gambiae* complex^79^. We therefore consider the most parsimonious argument for the evolution of anthropophily in the Leucosphyrus Subgroup to be that it evolved once only through the process of adaptive introgression at nodes N1/N2 as this accommodates the multiplicity of genes underlying the trait and negates any need to invoke loss of this trait. According to this hypothesis, anthropophily would have evolved between the extremes of the N1 and N2 confidence intervals i.e. between 2.9 and 1.6 mya (Fig. 3) when all the lineages were in Sundaland, and prior to the divergence of the Dirus Clade (node N3, 1.6 Mya [95% CI: 2.0–1.2 Mya]) further north in Indochina. This hypothesis could be tested against the above alternatives by identifying the genes underlying anthropophily and characterizing their evolutionary history.

Dating of the evolution of anthropophily in the Leucosphyrus Group to 2.9–1.6 mya overlaps with the earliest proposed date for the arrival of early hominins (*Homo erectus*) into Sundaland at 1.8 Mya^34^, but not with the more recent proposed date of 1.3 Mya^33^. Our findings suggest that anthropophily in the Leucosphyrus Group emerged in Sundaland in the early Pleistocene in response to the arrival of early hominins who must have not only been present in this region by this time but must have been in substantial numbers to drive adaptation to human host preference. This supports the hypothesis of Husson et al.^34^ that early hominins were present and abundant in Sundaland ~ 1.8 Mya, prior to their dispersal via land bridges to Java. Middle Pleistocene fossils of *Homo erectus* indicate their prolonged occupation on the exposed Sundaland landmass, likely associated with extensive river systems^83^. In the context of the very fragmentary nature of the fossil record in tropical SE Asia our findings contribute an important piece of evidence to the broader puzzle of the colonization of hominins in insular Southeast Asia.

## Materials and methods

### Fieldwork and sampling/Taxon sampling

Mosquito specimens (*n* = 38) were used to represent 11 of the 20 species in the Leucosphyrus Group, including species belonging to each Subgroup and representing all blood-feeding behaviours (Supplementary Table S2). Except for colony material of *An. cracens*, all the specimens were obtained as either larvae or adults from field collections between 1995 and 2020 (Supplementary Table S2). Most adult mosquitoes were collected using human landing catch, while *An. pujutensis*, *An. macarthuri*, *An. hackeri*, *An. introlatus* and one *An. balabacensis* and one *An. baimaii* were collected as larvae (Supplementary TableS2 ). The larvae were reared to adults for morphological identification to species or as belonging to the *An. dirus* complex using keys by Reid^13^ and E L Peyton^17^. Members of the *An. dirus* complex were identified to species based on ITS2 sequence data^84^.

### DNA extraction, library Preparation and sequencing

DNA extractions of whole mosquitoes were carried out following a phenol-chloroform protocol. Total genomic DNA extraction for a few mosquito specimens was performed with DNeasy Blood and Tissue Kits (Qiagen^®^), digested overnight with proteinase K following the manufacturer’s protocol, and eluted with elution buffer to either 50–100 µl. Extracted genomic DNA was quantified with a Qubit 4.0 fluorometer (Invitrogen, Carlsbad, CA, USA) using the manufacturer’s protocol and 20 µl of DNA (10-100ng/µl) per sample was sequenced at the Earlham Institute (Norwich, UK). Low Input, Transposase Enabled (LITE) libraries were constructed and sequenced on two lanes of the NovaSeq 6000 SP flow cell with 150 bp paired-end reads.

### Quality control of Raw reads

With a sequencing depth of ~ 30X, we were able to generate 3 million paired-end DNA sequence reads and roughly 10 GB of data per sample. To minimise sequencing errors several quality control steps were taken to filter out low-quality sequencing reads. Raw FASTQ reads were filtered using the software TrimGalore v0.6.7 developed by Babraham Bioinformatics^85^ by trimming adapters and low-quality bases (Phred quality ≥ 30) and cleaned sequences were reanalysed using FastQC^86^ and MultiQC (Babraham Bioinformatics).

### Single-copy ortholog identification and filtering

The assembled and annotated proteomes of the reference species *An. dirus s.s.* (ENA accession-GCA_000349145.11.1) from Thailand and an outgroup species *An. farauti* (ENA accession-GCA_000473445.2) from Papua New Guinea were used to define groups of orthologous sequences using OrthoFinder2^87^. Only single-copy orthologs (SCOs) that were present in all individuals and had a minimum length of 300-bp were chosen for downstream analysis, which resulted in 5,867 nuclear SCO protein-coding genes.

### Assembly, alignment and filtering of nuclear SCOs

We used aTRAM 2.0^88^, an iterative assembler that executes reference-guided local *de novo* assemblies, to assemble 5,867 SCOs from 38 genomes we sequenced. To do this, we used trimmed sequence reads that were first converted to a Blast database using the atram_preprocessor.py command of aTRAM v2.3.4. The amino acid sequences of 5,867 genes were used in tblastn along with the SPAdes assembler^89^, with five iterations, to create the aTRAM assemblies (atram.py script). The exon sequences from aTRAM assemblies were stitched together in the correct frame using the find_orthologs.py wrapper script^90^. Two publicly available genomes, one for *An. dirus s.s.* (GCA 000349145.1, Thailand)^37^ and one for *An. cracens* (GCA 002091845.1, peninsular Malaysia)^38^, were used in addition to the 38 novel genomes for phylogenetic analysis. From the 5,867 genes that were assembled by aTRAM we selected 2,928 that were present in all the samples for phylogenetic analysis. Each gene was aligned using the – auto flag in MAFFT^91^ and individual gene alignments were trimmed using the -automated1 flag in trimAl v. 1.4 0.1^92^. Gene alignment of length > 500 bp was chosen for phylogenetic analysis. This filtering resulted in 2,657 SCOs.

### Mitochondrial genome assembly and alignment

The mitochondrial genome was assembled using MITObim v1.9.1 program^93^. First, trimmed paired-end reads were interleaved using NGmerge v0.3^94^. The complete mitochondrial genome from *An. dirus s.s*. (GenBank accession NC_036263) was used as a reference seed, and the assemblies were created with 30 iterations and --quick option using the Mitobim.pl script. The FASTA reads retrieved in the final iteration were annotated using default parameters in the MITOS web server^95^ (http://mitos.bioinf.uni-leipzig.de/). The GFF files generated by MITOS were imported to Geneious Prime v11.0.4. and 13 mitochondrial protein coding genes (PCGs) were extracted from mitogenomes. Two published mitogenomes of *An. dirus s.s.* NC_036263 (Hainan, China) and *An. cracens*, NC_020768 (Thailand), were also used along with 38 samples. Geneious v11.0.4’s MUSCLE aligner was used to align PCGs from 40 individuals, and trimming was performed so that all genes were the same length.

### Sample validation

To validate the species identity and identify any potential contamination in our assembled sequences, we used the NCBI BLAST web interface to compare our ITS2 sequences assembled in aTRAM against the GenBank database. We also verified the *COI* sequences assembled using MITObim against BOLD and GenBank databases. Specifically, rDNA *ITS2* was used to distinguish *An. dirus* from *An. baimaii* as they cannot be separated using mtDNA *COI*^41^. Morphological identification for *An. hackeri*, *An. pujutensis*, *An. macarthuri*, *An. balabacensis* was performed in the field by an expert taxonomist (Ralph E. Harbach).

### Phylogenetic analysis

Two datasets were used to reconstruct the phylogeny of eleven species in the Leucosphyrus Group: mitochondrial PCGs and nuclear SCOs of 38 individuals and two reference genomes. Both concatenation and coalescent-based methods were used. We first concatenated all 13 PCGs in Geneious to make a bigger alignment file consisting of 9,900 bp. The nuclear dataset was made by concatenating 2,657 SCOs using the script concatenate.rb^90^ to make a supermatrix of 4,929,412 bp. Maximum likelihood phylogenetic reconstruction using the concatenated dataset was performed in IQ-TREE v2.1.2^40^. We ran MODELFINDER^96^ in IQ-TREE with the –m MFP option to find the best model. The best-fit model: TIM2 + F + I + G4F was chosen for mitochondrial PCGs and GTR + F + R10 was chosen for the nuclear dataset according to Bayesian Information Criterion. Incorporating these models in the respective datasets, 100 ultrafast bootstraps were performed in IQ-TREE v2.1.2. For the coalescent-based tree reconstruction, we first generated gene trees for each gene using 100 rapid bootstrap replicates in RAxML v8.2^97^ using a GTRGAMMA model. The gene trees for both the mitochondrial and nuclear datasets were then summarised using ASTRAL v5.7.8. Both the trees were visualised using iTOL^98^.

### Divergence time Estimation

Chronograms for nSCOs were generated using StarBeast3 which infers species trees from gene trees under the multispecies coalescent (MSC) model taking into account incomplete lineage sorting and/or introgression^46,99^. However, due to the computational demands of the Bayesian approach, we were unable to make use of the entire nuclear dataset. We used gene selection in SortaDate^47^ to create a reduced dataset that could be processed with the available computing power and time. In a study by Jarvis et al.^100^, “clock-like” genes were found to evolve at a steady rate and reduce errors caused by model misspecification^47^. The SortaDate filtering procedure required two primary inputs: a species tree topology and rooted gene trees. The species tree was constructed using all the loci with RAxML^97^, serving as a reference for comparison. Individual gene trees were rooted using the outgroup *An. macarthuri*. This species was selected as the outgroup based on its position in phylogenetic trees when a much more distant outgroup, *An. farauti*, was used (data not shown). SortaDate evaluates each gene tree based on three criteria; (i) clock-likeness assessed using a root-to-tip variance statistic, indicating how consistent the gene’s evolutionary rate is across lineages, (ii) topological similarity/bipartition support measured by how closely the gene tree’s topology matches the provided species tree, and (iii) tree length evaluated by the total branch length of the gene tree, reflecting the amount of evolutionary information it contains. The genes were then ranked based on bipartition support, root-to-tip variance and tree length. Prioritizing bipartition support first ensures that the selected genes are topologically congruent with the species tree, minimizing topological conflicts. Following this with root-to-tip variance emphasizes the selection of genes that exhibit clock-like behaviour, which is crucial for accurate molecular dating. Tree length is considered last, and it helps to avoid overemphasis on genes with high evolutionary rates that may not be suitable for divergence-time estimation^47^. Using this approach we selected 25 clock-like nSCO nucleotide alignments to create a dataset for divergence dating and trait analysis.

Divergence times were estimated using the mutation rate derived from spontaneous mutations in whole genomes of *Drosophila—*2.8 × 10^− 9^ mutations per site per generation with 11 generations per year^51–54^. To account for only protein coding regions being used here, this mutation rate was scaled by 0.524 based on the relative mutation rate of coding to non-coding autosomal regions in *Anopheles*^52^ to yield a rate 1.47 × 10^− 9^ mutations per site per generation for the coding data used here.

The optimal substitution model, yielding GTR + 4 gamma count categories, was inferred using bModelTest^101^ in BEAST v2.6.7. Additionally,.xml configuration files with a strict clock and relaxed clock were generated using the template StarBeast3 in BEAUti v2.6.7. Two independent MCMC runs were carried out, each with a chain length of 100,000,000 and logging every 50,000 generations, until the effective sample size reached above 200. Divergence time were also estimated using mitochondrial *CO1*gene. The.xml configuration file was generated using relaxed clock in standard Beast template with GTR (+ I and + G) model. The MCMC runs were carried out with a chain length of 10, 000,000 and logging every 1000 generation until the effective sample size reached above 200. To determine whether each run converged, we used Tracer v1.7.2 to visually evaluate traces and effective sample sizes for posteriors, and likelihoods. We used the TreeAnnotator application in BEAST v2.6.7^46^ to generate a maximum clade credibility tree. Trees were visualised in FigTree v1.4.4^102^.

### Ancestral state reconstruction

To infer historical biogeography of the Leucosphyrus Group including the relative roles of vicariance and dispersal, the ancestral states were reconstructed on a phylogenetic tree with the RASP (Reconstruct Ancestral State in Phylogenies) v4 software^57^ using Bayesian Binary MCMC (BBM) analysis. The Bayesian tree generated for divergence dating estimation in BEAST was used for this analysis. The geographical distributions of the Leucosphyrus Group species (Supplementary Table S3 ) were based on the literature^17^ (Supplementary Table S1) with each species assigned to one or more of the following eight geographical areas that capture biogeographical and landmass transitions: (A) Borneo, (B) Peninsular Malaysia + south Thailand below the Kangar-Pattani Line (K-PL), (C) from K-PL northwards to the Isthmus of Kra (ISK), (D) from ISK to northwestern Thailand along its border with Myanmar, (E) remaining area of Thailand + Laos + Vietnam + Cambodia, (F) Myanmar + northeast India, (G) Java, and (H) Sumatra. RASP analysis was also performed to track evolution of the trait for host preference in the Leucosphyrus Group. For this, three categories for trait state were used: (A) non-human primate feeders, (B) mostly anthropophilic, with a third category (AB) indicating feeding on both humans and NHPs without a strong preference, based on evidence from the literature outlined in Supplementary Table S1. For both historical biogeography inference and host preference trait analysis, the MCMC chains of the BBM analysis were run for one million generations, with a sampling frequency of every 100 generations and a 10% burn-in. A fixed JC + G (Jukes-Cantor + Gamma) model was used for the BBM analysis.

## Supplementary Information

Below is the link to the electronic supplementary material.

Supplementary Material 1

## Data Availability

All the trimmed genome sequence data are deposited in the NCBI SRA, BioProject ID [PRJNA1148154](https:/dataview.ncbi.nlm.nih.gov/object/PRJNA1148154?reviewer=8qu6nucnqaj8ihg744ttf0b07d). All the data files, scripts, and codes used in this study are deposited in Figshare [https://figshare.com/s/e82d3503a201e019eca7](https:/figshare.com/s/e82d3503a201e019eca7).

## Declarations

### Disclaimer

The opinions or assertions contained herein are the private views of the authors, and are not to be construed as official, or as reflecting true views of the US National Institutes of Health, US Department of the Army, US Department of Defense, or the US Government. The material in this manuscript has been reviewed by the Walter Reed Army Institute of Research. There is no objection to its presentation and/or publication.
