# Supplementary material for: Early hominin arrival in Southeast Asia triggered the evolution of major human malaria vectors
Source: Sci Rep. 2026 Feb 26;16:6973. doi: 10.1038/s41598-026-35456-y (PMC12946254; doi:10.1038/s41598-026-35456-y)
Supplement: Supplementary file 1 — Supplementary Material 1 [file 41598_2026_35456_MOESM1_ESM.docx]

**Supplementary materials**

Early hominin arrival in Southeast Asia triggered the evolution of major human malaria vectors

Upasana Shyamsunder Singh^1,2*†^, Ralph E. Harbach^3^, Jeffery Hii^4^, Moh Seng Chang^5^, Pradya Somboon^6^, Anil Prakash^1,7,8^, Devojit Sarma^1,7,8^, Ben S. Broomfield^1^, Katy Morgan^1^, Sandra Albert^9^, Aparup Das^10^, Yvonne-Marie Linton^11,12,13^, Jane M. Carlton^14^, Catherine Walton^1*^

Introgression analysis

Using PhyloNet v3.8.0 [1], we reconstructed phylogenetic networks that allow for reticulation between branches. The input comprised of gene trees with one representative individual per species. PhyloNet can infer species networks based on topology alone however, simulations have demonstrated that the accuracy of the inferred species networks can be enhanced by incorporating branch lengths. While using branch lengths, PhyloNet also requires the tree to be ultrametric therefore, input trees were generated in BEAST2 [2], which produces ultrametric trees, with branch lengths. The gene trees were estimated for 2,657 SCO using the R package babette [3] specifying the HKY model of sequence evolution and a chain length of 500,000 iterations. The R script convert_to_newick.r [4] was used to convert the Maximum clade credibility trees produced by BEAST2 for each SCO alignment into Newick format, to combine them into a single input file for PhyloNet. Finally, network searches were performed using “InferNetwork_ML” using branch lengths of gene tree (-bl), allowing 0–3 reticulations and optimising branch lengths and inheritance probabilities (-o). Five iterations were carried out and the log probability of the best inferred network was used to compare log probabilities for 1–3 reticulations (Supplementary Fig. S1) using a log likelihood ratio test. All the networks were visualized using Dendroscope3 [5]. By comparing the fit of models with increasing number of reticulations, we assessed whether the inclusion of additional reticulations significantly improved the model fit. A low p-value (typically < 0.001) suggests that the added reticulation significantly enhances the fit in our data.


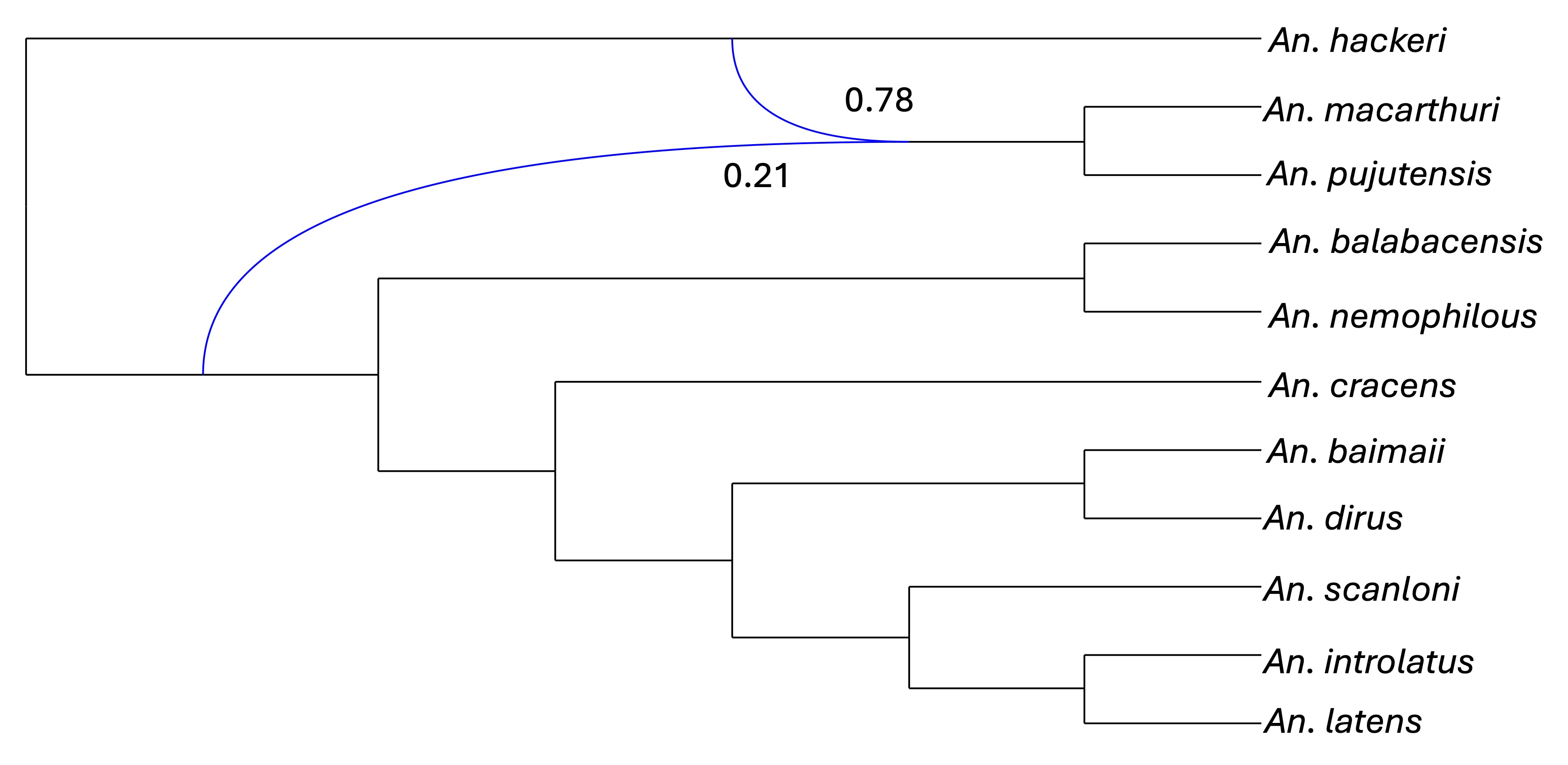


a.


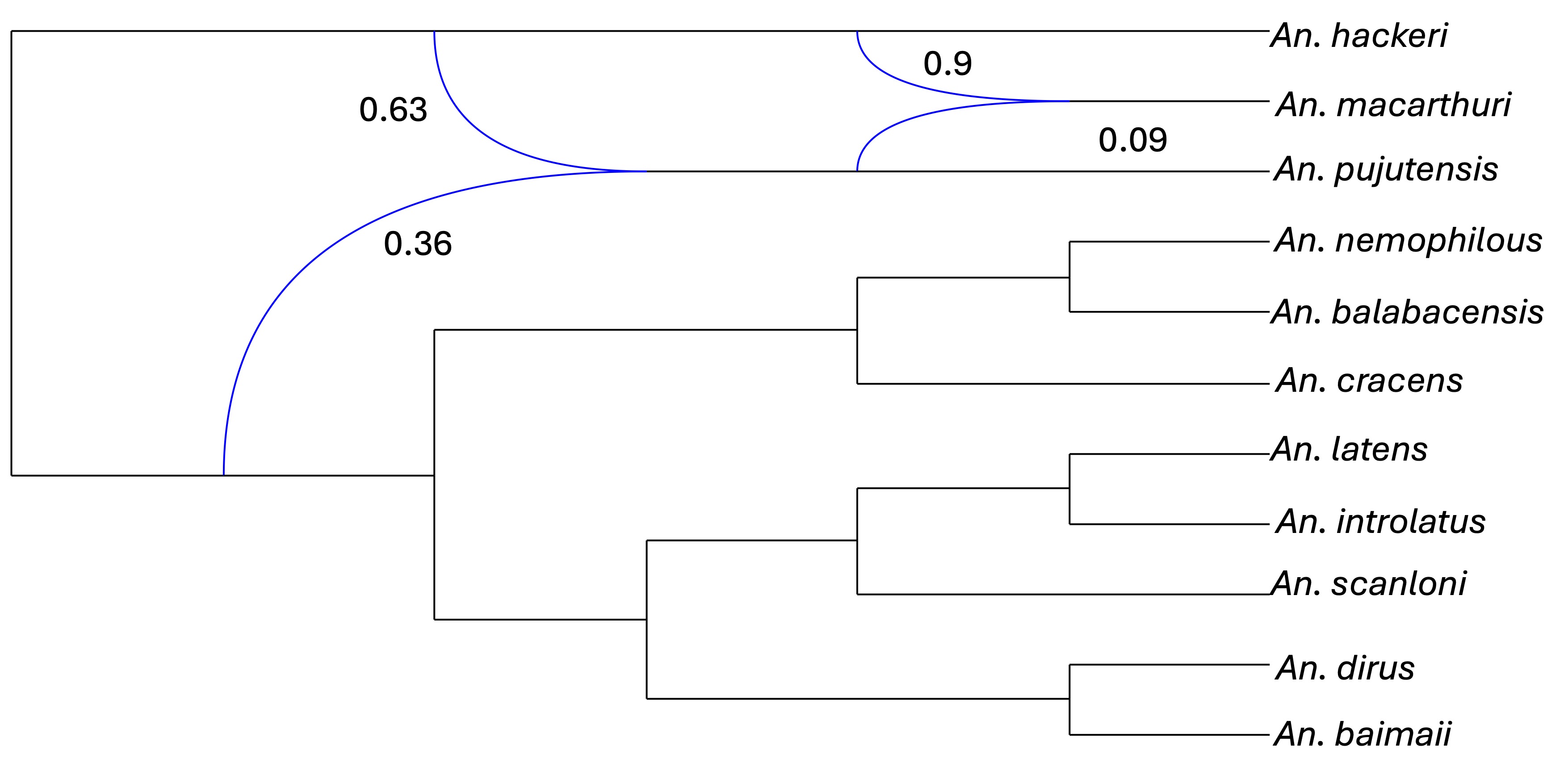


b.


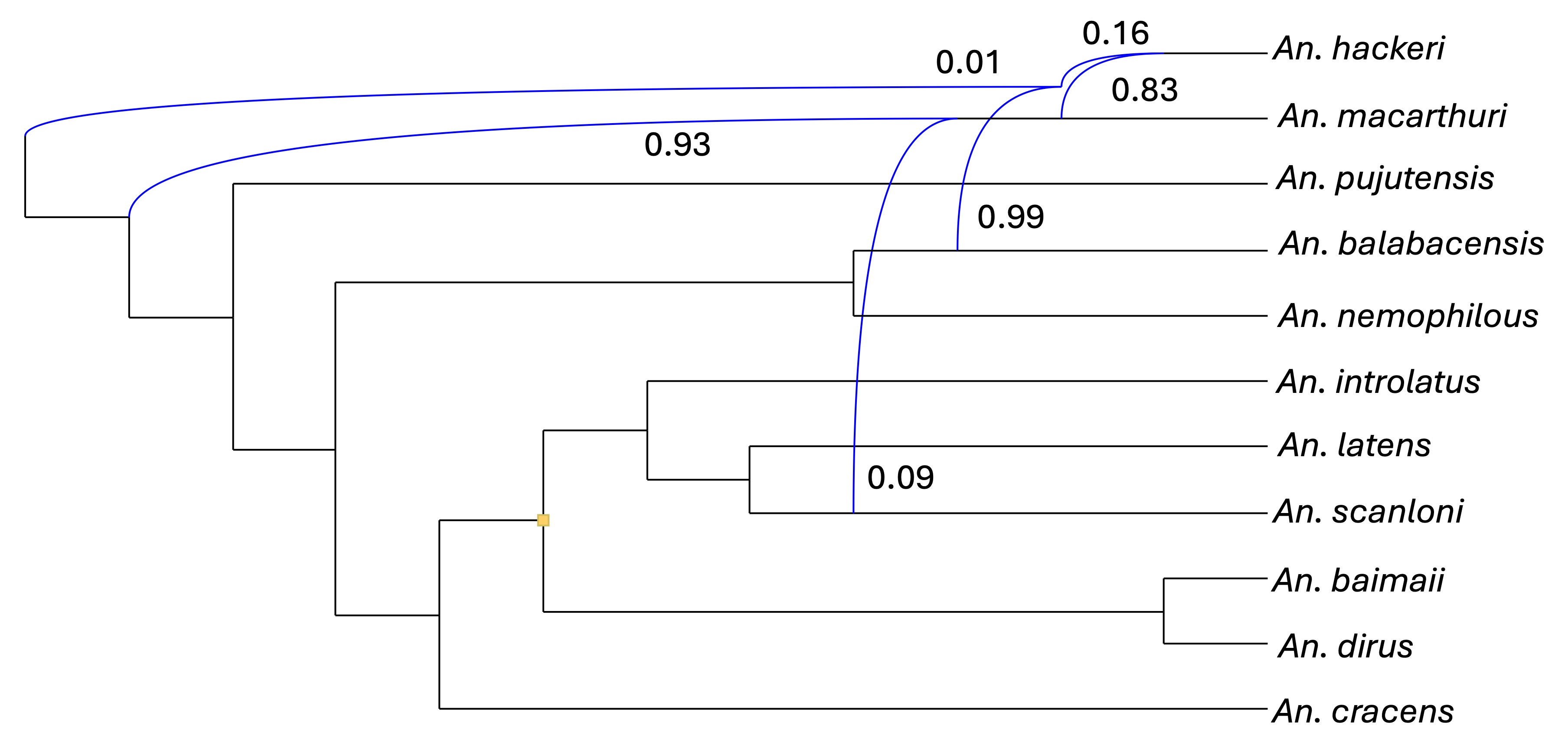


c.

**Figure S1**. **Phylogenetic networks inferred using maximum likelihood in PhyloNet.** Optimal networks A, B and C were inferred when the number of reticulations was set to 1, 2 and 3, respectively. The log-likelihoods of the three networks are -1733.79 (**a**), -1727.66 (**b**), and -1715.53 (**c**) with p< 0.001. The backbone network is depicted in black solid lines. The reticulation edges are shown in blue lines. The numbers adjoining reticulation nodes are inheritance probabilities.


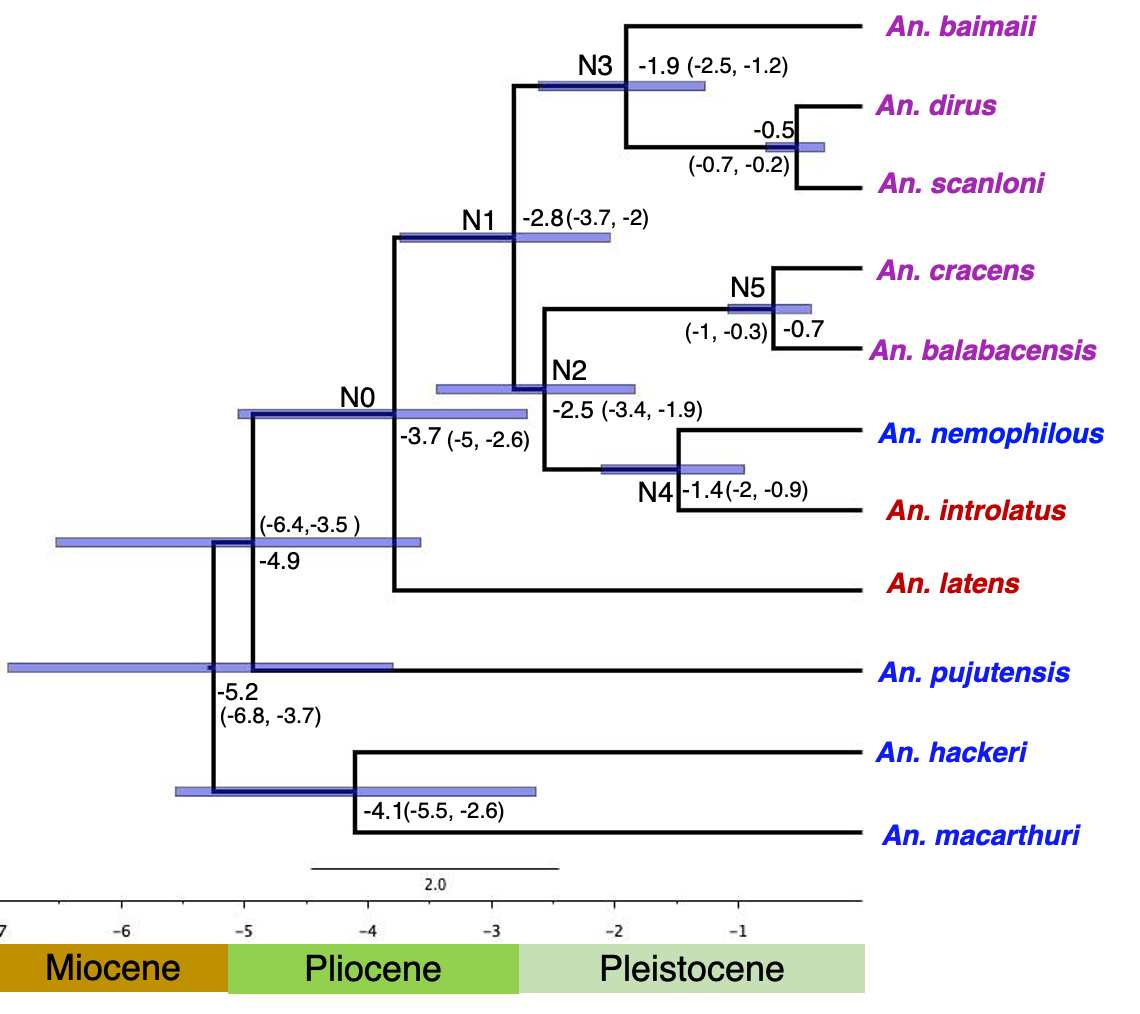

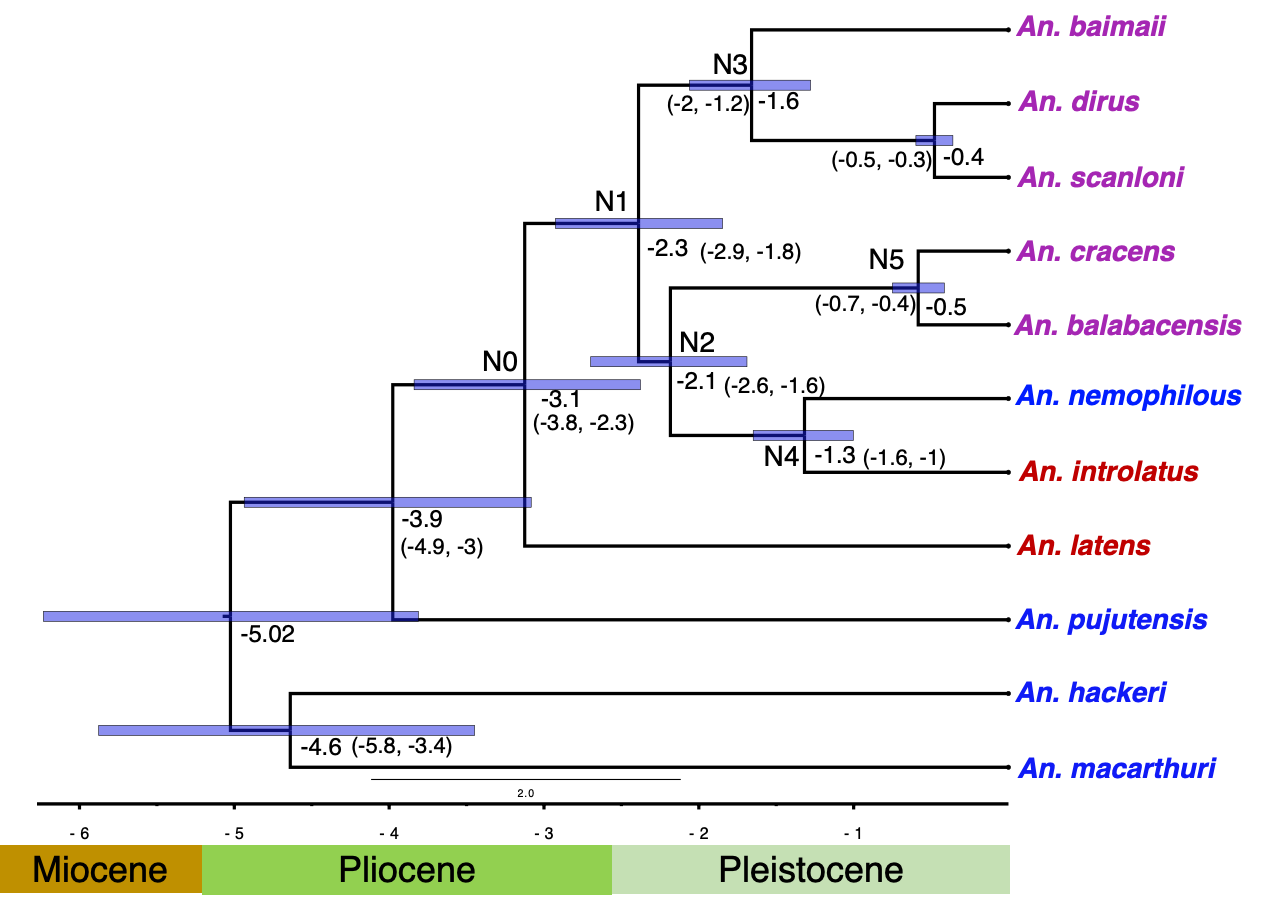


**Figure S2. Nuclear chronogram of the Leucosphyrus Group using a Bayesian approach**. The divergence dating analysis using 25 clock-like genes both strict clock (on the left) and relaxed clock (on the right) model. The estimated divergence times are indicated by the numbers at the nodes; the blue error bars denote 95% highest posterior densities, and the numbers in parentheses are confidence intervals. The colour of the species names indicates distinct blood-feeding behaviours; blue–NHP feeding, red–mixed-feeding, purple–human feeding.


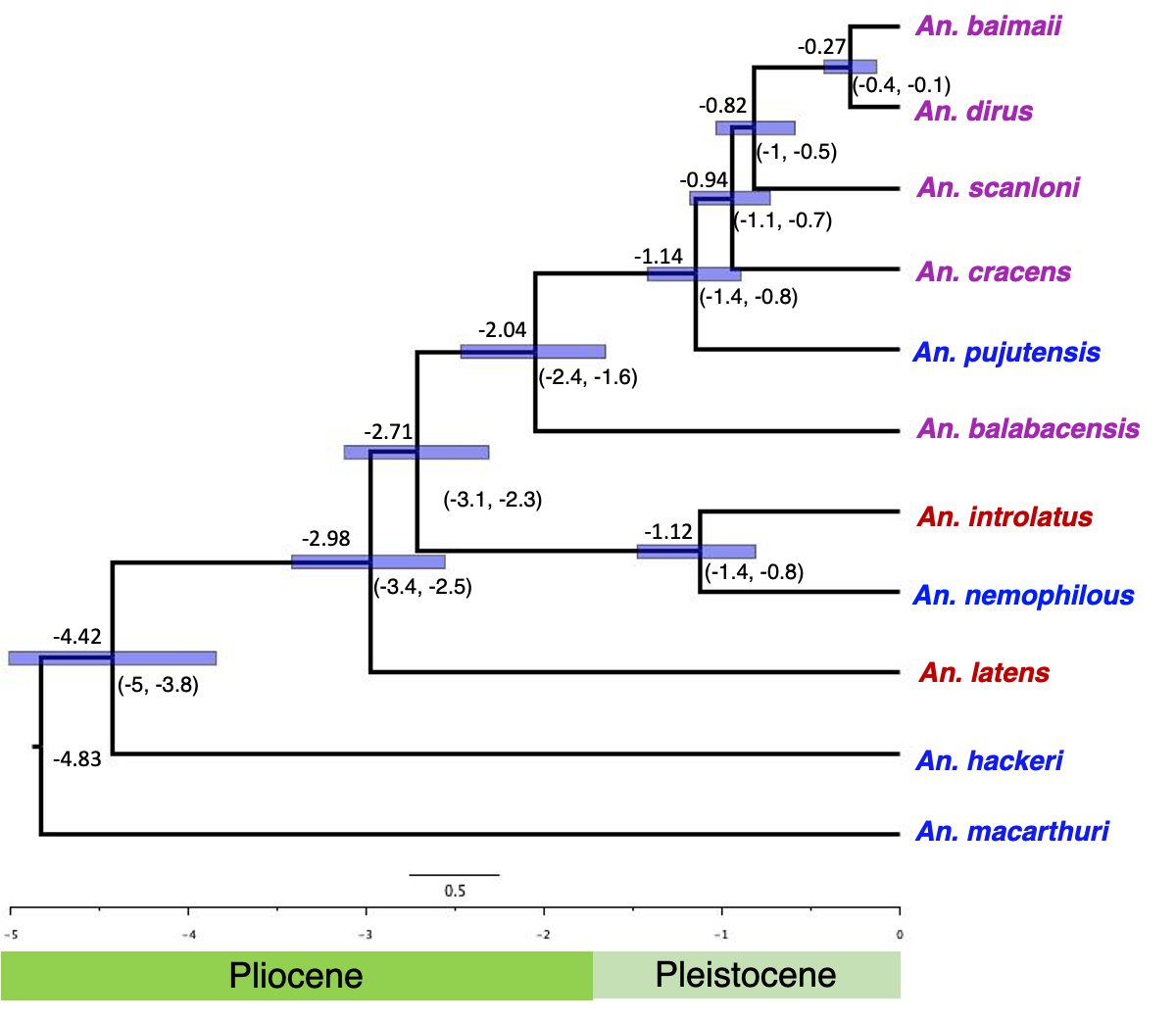


**Figure S3. Mitochondrial chronogram of the Leucosphyrus Group using a Bayesian approach**. The divergence dating analysis using the mitochondrial *COI* gene and relaxed clock model. The estimated divergence times are indicated by the numbers at the nodes; the blue error bars denote 95% highest posterior densities, and the numbers in parentheses are confidence intervals.

**Table S1.** **Evidence base for host feeding preferences of species of the Leucosphyrus Group used in this study.** The table compiles information from studies that used monkey and human baits variously deployed in the canopy and on the ground to study the feeding behaviour/host preference of Leucosphyrus Group mosquitoes. It also includes comments relevant to host preference made by observations during field collections of mosquitoes used in this study. The last column indicates the feeding category into which species were placed based on this evidence as used in the text and analysis of host preference trait evolution in RASP v4 (Reconstruct Ancestral State in Phylogenies). The three categories are: (A) strictly NHP feeders in the canopy; (B) highly anthropophilic behaviour at ground level and AB- host attraction for both NHP and humans. “Monkey feeding behaviour” is inferred here primarily from host choice experiments that were conducted using monkeys as bait (monkey bait traps [MBT]) however, as an extension we also imply host attraction (for blood feeding) to other NHPs in the forest of Southeast Asia such as gibbons and orangutans. Infection with monkey malarias (*P. knowlesi*, *P. cynomolgi*, *P. inui*, *P. coatneyi* and *P. fieldi*) is taken to indicate feeding on NHPs. HLC refers to human landing catches and where substantive numbers are caught indicates a preference for feeding on humans rather than NHPs in the canopy. Mosquito species names have changed over time as more species were discerned. While quotes from papers use the old name, the new name is indicated immediately afterwards in square brackets, where this can be reliably inferred.

| **Species** | **Feeding preference from the literature (References)** | **Comments from mosquito collectors (CW, REH)** | **Mosquito feeding group** |
| --- | --- | --- | --- |
| ***An. hackeri*** | Distinct preference for monkey bait and host seeking in the canopy [6, 7, 9, 10, 11, 12, 13]. The adults of *An. hackeri* and *An. pujutensis* were mostly caught on monkey bait in the canopy and parasite incrimination studies have confirmed that they are vectors of NHP malaria parasites. | Collected as larvae deep in forest in animal wallow | A |
| ***An. pujutensis*** |  | Collected as larvae only in forests | A |
| ***An. macarthuri***  (formerly *An. riparis macarthuri*) | Adults of *An. macarthuri* were only collected on monkey bait in the canopy in primary hill forests [6, 8, 11, 13]. | Strict NHP feeding indicated as mosquitoes were only collected in the larval state in this study (Sarawak, Sabah) but not in synchronous human baited collections in the same area | A |
| ***An. nemophilous***  (formerly *An. balabacensis* Fraser’s Hill form or *An. dirus* species F) | Baimai et al. [14] found that this species feeds primarily above ground level in the forest canopy. It was collected using human bait 12 m above ground level therefore, the author of this paper considers that it could be feeding on monkeys or mammals resting in the forest canopy, where humans are not found [14]. Peyton and Ramalingam [15] commented that this species is much more sensitive to forest cover than *An. dirus* which may relate to availability of NHP hosts. | Collected extremely rarely in human bait collections in Thailand despite *An. dirus* and *An. baimaii* being abundant in the same collections | A |
| ***An. latens***  (formerly referred to as *An. leucosphyrus* in Borneo and peninsular Malaysia) | Using human bait only, Baimai et al. [14] collected 37 mosquitoes on a platform, one on the ground in the forest and 32 outside houses in the village, implying that they feed both on the ground and in the canopy, on monkeys and humans.  In village settings (presumably no monkeys present), Colbourne et al. [16] noted, “*An. leucosphyrus* [*An. latens*] is anthropophilic: 78.8% fed on humans while the rest fed on dogs, pigs and fowls”.  Wharton et al [11] noted, “*A. leucosphyrus* [*An. latens*] and *A. b. introlatus* [*An. introlatus*] feed on monkeys in the canopy and bite man on the ground”.  In Sarawak, Malaysian Borneo, Tan et al. [13], noted: “*An. latens* was the only mosquito positive for sporozoites and it was found to be attracted to both human and monkey hosts. In monkey-baited net traps, it preferred to bite monkeys at the canopy level than at ground level.”  Eyles et al. [17] noted: “*An. leucosphyrus* [*An. latens*], does attack man and is considered a human malaria vector in some areas (Sarawak), but in Malaya does not attack man as readily as does *A. b. introlatus* [*An. introlatus*]”.  In a recent study, Jeyaprakasam et al [18] reported, “In a village and forest of Peninsular Malaysia, *An. latens* were infected with *P. fieldi* and *P. inui* (10% sporozoite rate).”, indicating feeding on NHP hosts. | Collected readily in human bait collections on the ground in Sarawak | AB |
| ***An. introlatus***  (formerly *An. balabacensis introlatus* or *A. b. introlatus*) | Isolations of *P. fieldi* [19] and *P. cynomolgi* [17] (monkey malaria parasites) were made from *An. introlatus* caught on human bait and monkey bait.    Wharton et al [11] noted: *An. leucosphyrus* [*An. latens*] prefers to feed in the canopy, whereas *A. b. introlatus* [*An. introlatus*] was caught in about equal numbers in the canopy on monkeys and at ground level on humans.”  According to Warren et al. [9] “*An. introlatus* readily entered the forest canopy to feed on monkeys and was almost equally prepared to invade human-baited net traps on the ground in the same area”. “*Anopheles balabacensis introlatus* [*An. introlatus*] and *Anopheles leucosphyrus* [*An. latens*] (both vectors of simian malaria) were trapped in the jungle with man as bait at ground level and monkey as bait in the forest canopy.”  Jeyaprakasam et al. [20] found that both *An. introlatus* and *An. cracens* feed on humans and monkeys based on blood meal analysis.  De Ang et al. [21] carried out human landing catches in the forest area where macaques live, and hunting is carried out by villagers. They found that *both An. introlatus* and *An. latens* are vectors of NHP malaria parasites in Sarawak with *An. introlatus* having salivary glands infected with *P. knowlesi, P. cynomolgi*, *P. fieldi*, *P. inui* and *P. coatneyi,* and *An. latens* with *P. knowlesi.*  Jeyaprakasam et al. [22] carried out human landing catches and light trap collections in forests and detected the NHP malaria parasites, *P. inui* and *P. fieldi* in *An. introlatus* and *An. cracens*.  Jeyaprakasam et al. [18] noted in the forests of Peninsular Malaysia, *An. introlatus* was predominantly collected using human landing catch and some of them were infected with *P. cynomolgi* and *P. inui*. This indicates that it bites both humans and NHPs. | Collected as larvae deep in the forest. | AB |
| ***An. cracens***  (formerly known as *An. balabacensis* Perlis Form and *An. dirus* species B) | Using human landing catches, Baimai et al. [14] collected, seven mosquitoes on a platform in the forest canopy, one mosquito on ground level in forest and nine mosquitoes in a village outside the forest suggesting that this species is highly anthropophilic.  *An. cracens* (as well as their sibling species *An. dirus*, *An. baimaii* and *An. scanloni*) were collected in substantive numbers using HLC [22].  A preference for biting humans is indicated by Sallum et al [23] who noted: “In Sabang Island (Pulau Weh), Aceh, Sumatra, *An. cracens* was captured biting humans at night (J. E. Hudson). In west Malaysia, adult females were also captured in human-bait collections at night in secondary tropical rain forest and in villages in localities situated either in mountainous or hilly areas.”  As reported above under *An. introlatus*, *An. cracens* was caught using HLC [22], indicating its anthropophilly but was also infected with NHP malaria parasites, *P. inui* and *P. fieldi*, indicating it also feeds on monkeys.  According to Jiram et al. [24], “*Anopheles cracens* was the predominant mosquito biting humans as well as the macaques. It comprised 63.2% of the total collection and was the only species positive for sporozoites of *P. knowlesi*.” | N/A – colony material only | B |
| ***An. balabacensis***  *(= An. balabacensis balabacensis)* | In Peninsular Malaysia “*An. balabacensis* preferred to bite man more than other animals” [25, 26]  Hii [27] noted, *An. balabacensis* is an extremely good vector of human malaria parasite and that it bites humans repeatedly while resting in houses.  Harbach et al. [28] noted based on HLC in 1986 in a remote village located in a heavily forested area of South Kalimantan, that “*Anopheles leucosphyrus* [*An. latens*] and *An. balabacensis* comprised 97.7% of the total number of specimens collected outside houses in the village” indicating both species readily feed on humans.  According to Hawkes et al. [29], “HLC caught more *An. balabacensis* than any other method (3.6 per night). In contrast, no *An. balabacensis* were collected in MBT collections” implying it likes humans.”  According to Brown et al. [30], “The primary *P. knowlesi* vector in Sabah, *An. balabacensis*, was detected at significantly higher numbers near trees with than without macaques; indicating this species is an acceptable host type”.  Brant et al. [31] noted, “the potential ability of *An.* *balabacensis* to transmit *P. knowlesi* between canopy-dwelling simian hosts and ground-dwelling humans”.  Wong et al. [32] in Sabah, detected *P. knowlesi* infection in *An. balabacensis* and noted: “*Anopheles balabacensis* mostly bites humans outdoors in the early evening between 1800 to 2000 hrs.” | Collected readily in human landing catches on the ground and in the canopy in Sabah | B |
| ***An. dirus***  (formerly known as *An. balabacensis* or *An. dirus* species A, from Thailand Cambodia, Lao PDR, Vietnam) | Using HLC*, An. dirus*, was primarily collected from forests [33, 34], the fringes of primary forests [35] and villages [34]. A high anthropophilic index was reported in all these studies at all the sites indicating that *An. dirus* prefers humans for blood feeding.  High anthropophilly is further indicated in *An. dirus* by numerous studies demonstrating its role as a primary vector with high levels of infection with human malarial parasites in Thailand [36-38], Cambodia [34], Laos [39-42] and Vietnam [34, 43].  *Anopheles dirus* is renowned for maintaining malaria transmission in areas even when numbers are low [39, 44] indicating repeated biting on humans. This is consistent with its high anthropophilic index of 0.69 [45].  Despite its strong preference for humans, *Plasmodium* parasites of NHP and human malarias have been detected by PCR [46–48] in salivary glands of *An. dirus*, indicating it is also willing to feed on NHPs. | Collected readily in human bait collections | B |
| ***An. baimaii***  (formerly known as *An. balabacensis* or *An. dirus* species D, from Myanmar, Bangladesh, India and Thailand) | *Anopheles baimaii* has been identified as a primary vector of human malaria parasites by multiple studies including in northeast India [49, 50], Bangladesh [51], Myanmar [52] and Thailand [53].  In northeast India, *An. baimaii* was found to have extremely high anthropophilic indices that were inferred from blood meal identifications [49, 50, 54].  In central Myanmar, *An. baimaii* was reported as a primary vector of human *Plasmodium* spp. and infected samples were collected using HLC indoors [52].  In a study conducted in Thailand, Tainchum et al. [53] noted: “In Mae Sot, *An. dirus* and *An. baimaii* had a preference/attraction to humans with 97% collected on human bait compared to cattle.”  Most collections of *An. baimaii* were conducted on the ground but Sallum et al. [23] indicated that it may also seek blood meals in the canopy noting: “In Thailand, adults of *An. baimaii* were collected biting humans in primary and secondary evergreen forest, primary and secondary deciduous forest, primary and secondary rain forest, secondary rain forest mixed with fruit plantations, orchards and rubber plantations, banana and pineapple plantations, villages, villages with fruit plantations, inside houses, and also in the canopy of rain forests 16.5 m above ground level”. | Collected readily in human bait collections | B |
| ***An. scanloni***  (formerly known as *An. balabacensis* or *An. dirus* species C) | *An. scanloni* was collected using HLC in villages surrounded by forest in Thailand [23, 55–57]*.* | Collected readily in human bait collections | B |

**Table S2. Details of mosquito samples, geographical locations and collection method used in the study.**

| **Sample no.** | **Species** | **Date of collection** | **Collection location** | **Latitude** | **Longitude** | **Method of collection** |
| --- | --- | --- | --- | --- | --- | --- |
| 1 | *An. baimaii* | 21/10/2006 | Myanmar, Taninthari, Dawei, Pakhet Village | 14.12330 | 98.32080 | Larval dipping |
| 2 | *An. baimaii* | 10/07/2001 | Thailand, Krabi Province, Plai Phraya, Ban Tally Ho | 8.62247 | 98.85439 | Human landing catch |
| 3 | *An. baimaii* | 15/10/2020 | India, Meghalaya, Garo hills, Gulpani Songmong | 25.20480 | 90.79050 | CDC- light trap |
| 4 | *An. baimaii* | 08/06/2004 | India, Assam, Dibrugarh, Jorajan | 27.34326 | 95.46376 | CDC-light trap |
| 5 | *An. baimaii* | 11/10/2006 | Myanmar, Rakhine, ButhiDaung, Tha Daw Village | 20.83087 | 92.41075 | Manual aspiration on cow |
| 6 | *An. baimaii* | 31/08/1997 | Thailand, Kanchanaburi, Hub-e-Teu | 14.38164 | 98.93928 | Human landing catch |
| 7 | *An. balabacensis* | 14/11/1996 | Malaysia, Sabah, Papar District, Lingan | 5.62819 | 116.00347 | Human landing catch |
| 8 | *An. balabacensis* | 30/11/1996 | Malaysia, Sabah, Papar District, Lingan | 5.62819 | 116.00347 | Human landing catch |
| 9 | *An. balabacensis* | 22/11/1996 | Malaysia, Sabah, Lahad Datu District, Danum Valley Conservation area | 4.96125 | 117.80786 | Larval dipping |
| 10 | *An. balabacensis* | 21/11/1996 | Malaysia, Sabah, Lahad Datu District, Borneo rainforest Lodge | 5.02519 | 117.75758 | Human landing catch |
| 11 | *An. balabacensis* | 30/11/1996 | Malaysia, Sabah, Papar District, Lingan | 5.62819 | 116.00347 | Human landing catch |
| 12 | *An. cracens* | 06/04/1992 | Peninsular Malaysia, Perlis, Padang Besar, US Army Station | 6.64918 | 100.27977 | Lab colony material |
| 13 | *An. dirus* | 27/08/1995 | Thailand, Tak Province, Mae Sot District, Ban Tam Suea | 16.71386 | 98.57806 | Human landing catch |
| 14 | *An. dirus* | 28/09/2002 | Thailand, Lampang, Ban Dung | 18.44681 | 99.79431 | Human landing catch |
| 15 | *An. dirus* | 04/12/2003 | Thailand, Kanchanaburi, Sai yok, Wang Krajae, Bon Bong Ti Noi | 14.26210 | 98.93090 | - |
| 16 | *An. dirus* | 16/10/1996 | Thailand, Sakhon Nakhon, Khok Si Suphan | 16.99861 | 104.30512 | Human landing catch |
| 17 | *An. dirus* | 30/10/2003 | Laos, Vientiane, Phon-Hong, Non Som Boon | 18.35013 | 102.27298 | Human landing catch |
| 18 | *An. hackeri* | 20/11/1996 | Malaysia, Sabah, Lahad Datu District, Danum Valley Conservation area | 4.96125 | 117.80786 | Larval dipping |
| 19 | *An. introlatus* | 20/11/1996 | Malaysia, Sabah, Lahad Datu District, Danum Valley Conservation area | 4.96125 | 117.80786 | Larval dipping |
| 20 | *An. latens* | 03/11/1996 | Malaysia, Sarawak, Kuching District, Chupak Village | 1.23122 | 110.43397 | Human landing catch |
| 21 | *An. latens* | 01/11/1996 | Malaysia, Sarawak, Kuching District, Chupak Village | 1.23122 | 110.43397 | Human landing catch |
| 22 | *An. latens* | 1997 | Malaysia, Sarawak, Song District, Song | 1.89856 | 112.59390 | Human landing catch |
| 23 | *An. latens* | 18/07/2001 | Thailand, Phang-nga, Mueang, Ban Song Prak | 8.60002 | 98.55164 | Human landing catch |
| 24 | *An. latens* | 17/07/2001 | Thailand, Songkhla, Sadao District, Padang Besar, Ban Kwon Kaow Haing | 6.70767 | 100.22986 | Human landing catch |
| 25 | *An. macarthuri* | 27/11/1996 | Malaysia, Sarawak, Miri District, Lambir National Park | 4.19567 | 114.04631 | Human landing catch |
| 26 | *An. macarthuri* | 03/11/1996 | Malaysia, Sarawak, Bau District, Tringgus | 1.25833 | 110.09619 | Larval dipping |
| 27 | *An. macarthuri* | 14/06/1998 | Peninsular Malaysia, Selangor, Ulu Gombak | 3.33131 | 101.77392 | Larval dipping |
| 28 | *An. nemophilous* | 1995 | Peninsular Malaysia, Perlis, Padang Besar | 6.64918 | 100.27977 | human landing catch |
| 29 | *An. nemophilous* | 01/09/1997 | Thailand, Kanchanaburi, Hub-e-Teu | 14.38164 | 98.93928 | Human landing catch |
| 30 | *An. pujutensis* | 27/11/1996 | Malaysia, Sarawak, Miri District, Pujut | 4.40683 | 114.02531 | Larval dipping |
| 31 | *An. pujutensis* | 28/11/1996 | Malaysia, Sarawak, Miri District, Bukit Kigang | 3.63772 | 114.64264 | Larval dipping |
| 32 | *An. pujutensis* | 28/11/1996 | Malaysia, Sarawak, Miri District, Bukit Kigang | 3.63772 | 114.64264 | Larval dipping |
| 33 | *An. scanloni* | 31/08/1997 | Thailand, Kanchanaburi, Hub-e-Teu | 14.38164 | 98.93928 | Human landing catch |
| 34 | *An. scanloni* | 31/08/1997 | Thailand, Kanchanaburi, Hub-e-Teu | 14.38164 | 98.93928 | Human landing catch |
| 35 | *An. scanloni* | 22/10/1996 | Thailand, Nakhon Si Thammarat, Thung Song, Ban Nam Tok | 7.96083 | 99.75672 | Human landing catch |
| 36 | *An. scanloni* | 22/10/1996 | Thailand, Nakhon Si Thammarat, Thung Song, Ban Nam Tok | 7.96083 | 99.75672 | Human landing catch |
| 37 | *An. scanloni* | 22/10/1996 | Thailand, Nakhon Si Thammarat, Thung Song, Ban Nam Tok | 7.96083 | 99.75672 | Human landing catch |
| 38 | *An. scanloni* | 22/10/1996 | Thailand, Nakhon Si Thammarat, Thung Song, Ban Nam Tok | 7.96083 | 99.75672 | Human landing catch |

**Table S3**. **The current geographical ranges of members of the Leucosphyrus Group mosquitoes from the literature**

| **Species** | **Geographical range** |
| --- | --- |
| *An. macarthuri* | Peninsular Malaysia, Borneo, Thailand K-PL-ISK |
| *An. hackeri* | Borneo, Palawan, Peninsular Malaysia, Thailand K-PL-ISK |
| *An. pujutensis* | Sumatra, Peninsular Malaysia, Borneo, Thailand below K-PL |
| *An. balabacensis* | Palawan, (Sabah and Sarawak/Borneo), Java, Kalimantan, Brunei, |
| *An. latens* | Peninsular Malaysia, Borneo, Southern Thailand (below (ISK)) |
| *An. introlatus* | Peninsular Malaysia, Thailand below K-PL, Indonesia (in Sumatra), Borneo |
| *An. nemophilous* | Peninsular Malaysia, Thailand (K-PL-ISK), Northwestern Thailand, Thailand-central to north |
| *An. cracens* | Peninsular Malaysia, Thailand K-PL-ISK, Sumatra in Indonesia |
| *An. scanloni* | Northwestern Thailand, Thailand K-PL-ISK |
| *An. dirus* | Cambodia, Vietnam, Laos, Hainan-China, Thailand (central to north) |
| *An. baimaii* | Northeast India including Andaman and West Bengal, Myanmar, Bangladesh, Thailand (K-PL-ISK) + Northwestern Thailand |

**References :**

1. D. Wen, Y. Yu, J. Zhu, L. Nakhleh, Inferring phylogenetic networks using PhyloNet. *Systematic Biology* **67**, 735–740 (2018).
2. R. Bouckaert, T. G. Vaughan, J. Barido-Sottani, S. Duchêne, M. Fourment, A. Gavryushkina, J. Heled, G. Jones, D. Kühnert, N. De Maio, M. Matschiner, BEAST 2.5: An advanced software platform for Bayesian evolutionary analysis. *PLoS Computational Biology* **15,** e1006650 (2019).
3. R. J. C. Bilderbeek, R. S. Etienne, babette: BEAUti 2, BEAST2 and Tracer for R. *Methods Ecol. Evol*. **9**, 2034–2040 (2018).
4. M. Malmstrøm, M. Matschiner, O. K. Tørresen, B. Star, L. G. Snipen, T. F. Hansen, H. T. Baalsrud, A. J. Nederbragt, R. Hanel, W. Salzburger, N. C. Stenseth, Evolution of the immune system influences speciation rates in teleost fishes. *Nature Genetics* **48**, 1204–1210 (2016).
5. D. H. Huson, C. Scornavacca, Dendroscope 3: an interactive tool for rooted phylogenetic trees and networks. *Syst. Biol.* ***61*,** 1061–1067 (2012).
6. J. A. Reid, Anopheline mosquitoes of Malaya and Borneo. *Studies from the Institute for Medical Research Malaysia* **31**, 520 (1968).
7. R. H. Wharton, D. E. Eyles, *Anopheles hackeri*, a vector of *Plasmodium knowlesi* in Malaya. *Science* **134**, 279–280 (1961).
8. M. Warren, R. H. Wharton, Symposium on simian malaria. The vectors of simian malaria: identity, biology, and geographical distribution. *Journal of Parasitology* **49**, 892–904 (1963).
9. M. Warren, W. H. Cheong, H. K. Fredericks, G. R. Coatney, Cycles of jungle malaria in West Malaysia. *American Journal of Tropical Medicine and Hygiene* **19,** 383–393 (1970).
10. J. A. Reid, B. Weitz, Anopheline mosquitoes as vectors of animal malaria in Malaya. *Annals of Tropical Medicine and Parasitology* **55**, 180–186 (1961).
11. R. H. Wharton, D. E. Eyles, M. Warren, W. H. Cheong, Studies to determine the vectors of monkey malaria in Malaya. *Annals of Tropical Medicine and Parasitology* **58**, 56–77. (1964).
12. R. H. Wharton, D. E. Eyles, M. Warren, The development of methods for trapping the vectors of monkey malaria. *Ann. Trop. Med. Parasitol.* **57**, 32–46 (1963).
13. C. H. Tan, I. Vythilingam, A. Matusop, S. T. Chan, B. Singh, Bionomics of *Anopheles* *latens* in Kapit, Sarawak, Malaysian Borneo in relation to the transmission of zoonotic simian malaria parasite *Plasmodium knowlesi*. *Malaria Journal* **7**, 1–8 (2008).
14. V. Baimai, R. E. Harbach, U. Kijchalao, Cytogenetic evidence for a fifth species within the taxon *Anopheles dirus* in Thailand. *Journal of the American Mosquito Control Association* **4**, 333–338 (1988a).
15. E. L. Peyton, S. Ramalingam, *Anopheles (Cellia) nemophilous*, a new species of the Leucosphyrus Group from Peninsular Malaysia and Thailand (Diptera: Culicidae). *Mosquito Systematics* **20**, 272–289 (1988).
16. M. J. Colbourne, W. H. Huehne, F. S. LaChance, The Sarawak anti-malaria project. *Sarawak Museum Journal* **9**, 215–248 (1959).
17. D. E. Eyles, M. Warren, E. Guinn, R. H. Wharton, C. P. Ramachandran, Identification of *Anopheles balabacensis* introlatus as a vector of monkey malaria in Malaya. *Bull. World Health Organ.* **28***,* 134–135 (1963).
18. N. K. Jeyaprakasam, V. L. Low, S. Pramasivan, J. W. Liew, W. Y. Wan-Sulaiman, I. Vythilingam, High transmission efficiency of the simian malaria vectors and population expansion of their parasites *Plasmodium cynomolgi* and *Plasmodium inui*. *PLoS Neglected Tropical Diseases* **17**, e0011438 (2023).
19. D. E. Eyles, The species of simian malaria: taxonomy, morphology, life cycle, and geographical distribution of the monkey species. *The Journal of Parasitology* **49,** 866–887 (1963).
20. N. K. Jeyaprakasam, V. L. Low, J. W. Liew, S. Pramasivan, W. Y. Wan-Sulaiman, A. Saeung, I. Vythilingam, Blood meal analysis of *Anopheles* vectors of simian malaria based on laboratory and field studies. *Scientific Reports* **12**, 1–3 (2022).
21. J. X. De Ang, K. Yaman, K. A. Kadir, A. Matusop, B. Singh, New vectors that are early feeders for Plasmodium knowlesi and other simian malaria parasites in Sarawak, Malaysian Borneo. *Scientific Reports* **11**, 7739 (2021).
22. N. K. Jeyaprakasam, S. Pramasivan, J. W. Liew, L. Van Low, W. Y. Wan-Sulaiman, R. Ngui, J. Jelip, I. Vythilingam, Evaluation of Mosquito Magnet and other collection tools for *Anopheles* mosquito vectors of simian malaria. *Parasites & Vectors* **14**, 1–3 (2021).
23. M. A. Sallum, E. L. Peyton, B. A. Harrison, R. C. Wilkerson, Revision of the Leucosphyrus group of *Anopheles* (*Cellia*) *(Diptera, Culicidae)*. *Revista Brasileira de Entomologia* **49**, 1–152 (2005).
24. A. I. Jiram, I. Vythilingam, Y. M. NoorAzian, Y. M. Yusof, A. H. Azahari, M. Y. Fong, Entomologic investigation of *Plasmodium knowlesi* vectors in Kuala lipis, Pahang, Malaysia. *Malaria Journal* **11**, 1–10 (2012).
25. I. Vythilingam, G. L. Chiang, H. L. Lee, K. Singh, Special report on bionomics of important mosquito vectors in Malaysia. *Southeast Asian J. Trop. Med. Public Health* **23**, 581–602. (1992).
26. J. L. Hii, Y. Vun, The influence of a heterogeneous environment on host feeding behaviour of *Anopheles balabacensis* (Diptera: Culicidae). *Trop. Biomed* **4**, 67‒70 (1987).
27. J. L. Hii, Evidence for the existence of genetic variability in the tendency of *Anopheles balabacensis* to rest in houses and to bite man. *Southeast Asian J. Trop. Med. Public Health* **16,** 173‒182 (1985).
28. R. E. Harbach, V. Baimai, S. Sukowati, Some observations on sympatric populations of the malaria vectors *Anopheles leucosphyrus* and *Anopheles balabacensis* in a village-forest setting in South Kalimantan. *Southeast Asian Journal of Tropical Medicine and Public Health* **18**, 241–247 (1987).
29. F. Hawkes, B. O. Manin, S. H. Ng, S. J. Torr, C. Drakeley, T. H. Chua, H. M. Ferguson, Evaluation of electric nets as means to sample mosquito vectors host-seeking on humans and primates. *Parasites & Vectors* **10**, 1–3 (2017).
30. R. Brown, M. Salgado-Lynn, A. Jumail, C. Jalius, T. H. Chua, I. Vythilingam, H. M. Ferguson, Exposure of primate reservoir hosts to mosquito vectors in Malaysian Borneo. *EcoHealth* **19**, 233–245 (2022).
31. H. L. Brant, R. M. Ewers, I. Vythilingam, C. Drakeley, S. Benedick, J. D. Mumford, Vertical stratification of adult mosquitoes (Diptera: Culicidae) within a tropical rainforest in Sabah, Malaysia. *Malaria Journal* **15**, 1–9 (2016).
32. M. L. Wong, T. H. Chua, C. S. Leong, L. T. Khaw, K. Fornace, W. Y. Wan-Sulaiman, T. William, C. Drakeley, H. M. Ferguson, I. Vythilingam, Seasonal and spatial dynamics of the primary vector of *Plasmodium knowlesi* within a major transmission focus in Sabah, Malaysia. *PLoS Neglected Tropical Diseases* **9**, e0004135 (2015).
33. É. A. Vajda, A. Ross, D. Doum, E. L. Fairbanks, N. Chitnis, J. Hii, S. J. Moore, J. H. Richardson, M. Macdonald, S. Sovannaroth, P. Kimheng, Field evaluation of a volatile pyrethroid spatial repellent and etofenprox treated clothing for outdoor protection against forest malaria vectors in Cambodia. *Scientific Reports* **14**, 17348 (2024).
34. J. Hii, J. Hustedt, M. J. Bangs, Residual malaria transmission in select countries of Asia-Pacific region: old wine in a new barrel. *The Journal of Infectious Diseases* **223**, S111–S142 (2021).
35. A. Vantaux, M. M. Riehle, E. Piv, E. J. Farley, S. Chy, S. Kim, A. G. Corbett, R. L. Fehrman, A. Pepey, K. Eiglmeier, D. Lek, *Anopheles* ecology, genetics and malaria transmission in northern Cambodia. *Scientific Reports* **11**, 6458 (2021).
36. B. Greenwood, K. De Cock, Eds., *New and resurgent infections: prediction, detection and management of tomorrow’s epidemics*. (Chichester UK: John Wiley & Sons, 1998).
37. P. Singhasivanon, K. Thimasarn, S. Yimsamran, K. Linthicum, K. Nualchawee, D. Dawreang, S. Kongrod, N. Premmanisakul, W. Maneeboonyang, N. Salazar, Malaria in tree crop plantations in south-eastern and western provinces of Thailand. *Southeast Asian J. Trop. Med. Public Health* **30**, 399–404 (1999).
38. C. Tananchai, S. Manguin, M. J. Bangs, T. Chareonviriyaphap, Malaria vectors and species complexes in Thailand: implications for vector control. *Trends in Parasitology* **35**, 544-558 (2019).
39. H. D. Trung, W. Van Bortel, T. Sochantha, K. Keokenchanh, N. T. Quang, L. D. Cong, M. Coosemans, Malaria transmission and major malaria vectors in different geographical areas of Southeast Asia. *Trop. Med. Int. Health* **9**, 230–237 (2004).
40. I. Vythilingam, K. Keokenchan, S. Phommakot, S. Nambanya, S. Inthakone, Preliminary studies of *Anopheles* mosquitos in eight provinces in Lao PDR. *Southeast Asian J. Trop. Med. Public Health* **32**, 83–87 (2001).
41. T. Toma, I. Miyagi, T. Okazawa, J. Kobayashi, S. Saita, A. Tuzuki, H. Keomanila, S. Nambanya, S. Phompida, M. Uza, M. Takakura, Entomological surveys of malaria in Khammouane Province, Lao PDR, in 1999 and 2000. *Southeast Asian J. Trop. Med. Public Health* **33**, 532–546 (2002).
42. J. Kobayashi, S. Phompida, T. Toma, S. Looreensuwan, H. Toma, I. Miyagi, The effectiveness of impregnated bed net in malaria control in Laos. *Acta Tropica* **89**, 299–308 (2004).
43. H. M. Edwards, V. D. Chinh, B. Le Duy, P. V. Thanh, N. D. Thang, D. M. Trang, I. Chavez, J. Hii, Characterising residual malaria transmission in forested areas with low coverage of core vector control in central Viet Nam. *Parasites & Vectors* **12**, 1–6 (2019).
44. R. Rosenberg, R. G. Andre, L. Somchit, Highly efficient dry season transmission of malaria in Thailand. *Trans. R. Soc. Trop. Med. Hyg.* **84**, 22–28 (1990).
45. S. Marcombe, S. Maithaviphet, R. Reyburn, K. Kunlaya, K. Silavong, B. Hongvanthong, V. Vanisaveth, V. Sengsavath, V. Banouvong, K. Chindavongsa, B. Khamlome, Bionomics of malaria vectors in Lao PDR, 2018–2020: entomological surveillance as a key tool for malaria elimination. *Malaria Journal* **22**, 319 (2023).
46. S. Nakazawa, R. P. Marchand, N. T. Quang, R. Culleton, N. D. Manh, Y. Maeno, *Anopheles dirus* co-infection with human and monkey malaria parasites in Vietnam. *Int. J. Parasitol.* **39**, 1533–1537 (2009).
47. Y. Maeno, N. T. Quang, R. Culleton, S. Kawai, G. Masuda, S. Nakazawa, R. P. Marchand, Humans frequently exposed to a range of non-human primate malaria parasite species through the bites of *Anopheles dirus* mosquitoes in South-central Vietnam. *Parasites & Vectors* **8**, 1–7 (2015).
48. R. P. Marchand, R. Culleton, Y. Maeno, N. T. Quang, S. Nakazawa, Co-infections of *Plasmodium knowlesi, P. falciparum*, and *P. vivax* among Humans and *Anopheles dirus* Mosquitoes, Southern Vietnam. *Emerging Infectious Diseases* **17,** 1232 (2011).
49. A. Prakash, D. R. Bhattacharyya, P. K. Mohapatra, J. Mahanta, Indoor biting behaviour of *Anopheles dirus* Peyton and Harrison, 1979 in upper Assam, India. *Mosquito Borne Diseases Bulletin* **14**, 31–37 (1997).
50. A. Prakash, D. R. Bhattacharyya, P. K. Mohapatra, J. Mahanta, Malaria transmission risk by the mosquito *Anopheles baimaii* (formerly known as *An. dirus* species D) at different hours of the night in North‐east India. *Medical and Veterinary Entomology* **4**, 423–427 (2005).
51. M. H. Uddin, U. Habiba, M. R. Hamid, S. B. Rashid, M. A. Shahid, Vector Bionomics of Anopheline Fauna in Malaria Endemic Areas (Three Sentinel Sites) of Bangladesh. *Saudi J Med Pharm Sci*. **9**, 14–23 (2023).
52. W. Tun-Lin, M. M. Thu, S. M. Than, M. M. Mya, Hyperendemic malaria in a forested, hilly Myanmar village. *J. Am. Mosq. Control Assoc.* **11**, 401–407 (1995).
53. K. Tainchum, W. Ritthison, T. Chuaycharoensuk, M. J. Bangs, T. Chareonviriyaphap, Diversity of *Anopheles* species and trophic behavior of putative malaria vectors in two malaria endemic areas of northwestern Thailand. *Journal of Vector Ecology* **39**, 424–436 (2014).
54. P. Dutta, D. R. Bhattacharyya, S. A. Khan, C. K. Sharma, J. Mahanta, Feeding patterns of *Anopheles dirus*, the major vector of forest malaria in northeast India. *Southeast Asian J. Trop. Med. Public Health* **27**, 378–381 (1996).
55. S. R. Meek, Vector control in some countries of Southeast Asia: comparing the vectors and the strategies. *Ann. Trop. Med. Parasitol.* **89**, 135–147 (1995).
56. S. Manguin, P. Kengne, L. Sonnier, R. E. Harbach, V. Baimai, H. D. Trung, M. Coosemans, SCAR markers and multiplex PCR- based identification of isomorphic species in the *Anopheles dirus* complex in Southeast Asia. *Medical and Veterinary Entomology* **16**, 46–54 (2002).
57. S. M. O'Loughlin, P. Somboon, C. Walton, High levels of population structure caused by habitat islands in the malarial vector *Anopheles scanloni*. *Heredity* **99**, 31–40 (2007).
